# Noise induced unanimity and disorder in opinion formation

**DOI:** 10.1371/journal.pone.0235313

**Published:** 2020-07-09

**Authors:** Agnieszka Kowalska-Styczeń, Krzysztof Malarz

**Affiliations:** 1 Silesian University of Technology, Faculty of Organisation and Management, Zabrze, Poland; 2 AGH University of Science and Technology, Faculty of Physics and Applied Computer Science, Kraków, Poland; University of Catania, ITALY

## Abstract

We propose an opinion dynamics model based on Latané’s social impact theory. Actors in this model are heterogeneous and, in addition to opinions, are characterised by their varying levels of persuasion and support. The model is tested for two and three initial opinions randomly distributed among actors. We examine how the noise (randomness of behaviour) and the flow of information among actors affect the formation and spread of opinions. Our main research involves the process of opinion formation and finding phases of the system in terms of parameters describing noise and flow of the information for two and three opinions available in the system. The results show that opinion formation and spread are influenced by both (i) flow of information among actors (effective range of interactions among actors) and (ii) noise (randomness in adopting opinions). The noise not only leads to opinions disorder but also it promotes consensus under certain conditions. In disordered phase and when the exchange of information is spatially effectively limited, various faces of disorder are observed, including system states, where the signatures of self-organised criticality manifest themselves as scale-free probability distribution function of cluster sizes. Then increase of noise level leads system to disordered random state. The critical noise level above which histograms of opinion clusters’ sizes loose their scale-free character increases with increase of the easy of information flow.

## Introduction

Understanding how opinions are formed and spread in society is very important in studying consumer behaviour, organisational behaviour, predicting election results, and many others. As pointed out by Acemoglu and Ozdaglar [1], we acquire our opinions and beliefs in the process of social learning, during which people get information and update their opinions as a result of their own experience, as well as observation of other people’s activities and from their experience. This process takes place in a social network consisting of friends, co-workers, family members and a certain group of leaders that we listen to and respect [1, 2]. Units update and create their views by communicating with other people who belong to their social network. It is communication that connects people and creates relationships [3].

It should be noted that people often copy the choices of others [4, 5]. This applies, for example, to the choice of names for children [6, 7], a popular book, dishes ordered in a restaurant (instead of studying the menu, we look at what the others have ordered), and even ideological beliefs [5]. This copying of opinions and behaviours often takes place in a network of informal contacts and it is based on social relations between people [8] and plays an important role in forming opinions. In addition, we are often dealing with unpredictability or indifference in opinion-forming or decision-making (despite the positive attitude towards the proposed actions). This applies, among others, to electricity tariffs, eco-innovations or pro-environmental attitudes [9–11], as well as voting behaviour [12], in which human rationality is bounded. One of the most active discussions in psychology of the opinion dynamics is also about the irrational processing of information [13], therefore, this aspect should be taken into account in studying opinion formation. Furthermore, individuals belong to many groups, or have many interactions outside the main group (a group of closest neighbours). Such a connection with people from other groups (neighbourhoods) increases the information advantage [14], and can be interesting in disseminating information.

We therefore propose a model of forming an opinion based on the social impact theory formulated by Latané [15], in which we take into account the randomness of the actors’ behaviour by introducing a noise (social temperature), as well as interactions with agents; not only close neighbours, but in the whole network by *α* parameter (scale the distance function). Our agents are heterogeneous through a different level of persuasion intensity and support intensity, as well as the possibility of having different opinions.

Recently, the multi-choice opinion dynamics model [16, 17] based on Latané theory [15, 18–21] was proposed. In this model, it is possible to test the diffusion of opinions in case there are more than two opinions available in the system. The earlier attempts to modelling multiple-choice of opinions include among others multi-state and discrete-state opinions models [22–32] or discrete vector-like variables [33–36]. The rest of huge literature (see papers by Sîrbu et al., Castellano et al., Stauffer, Anderson et al., and Galam [37–41] for reviews) is devoted to the systems with binary opinions (see for example Refs. [42–45]) or the continuous space of opinions (see for example Refs. [13, 46–62]).

There are also many examples showing that randomness is useful and beneficial. Random noise facilitates the dynamics and reduces relaxation times in the models of social influence [63]. In addition, noise plays a beneficial role in developing cooperation [64], in the application of social and financial strategies [65] and in addressing the coordination problem of human groups [66].

In this paper we study how opinions are formed and how they spread in the community. Agent based model with lattice fully populated by actors has been adopted, where each of the lattice nodes refers to one person. We take into account the flow of information in the community (effective range of actors interactions) and noise (randomness of human behaviour). We show with computer simulation, that small level of noise induces unanimity of opinion. Unfortunately, this beneficial noise role was overlooked in Ref. [16] due to unreasonable extrapolation of results for middle noise level towards noiseless system.

## Model

To study the diffusion of opinions, the theory of social influence introduced by Latané [15] in the dynamic manner proposed by Nowak et al. [21]—as implemented by Bańcerowski and Malarz [16]—has been used.

Social influence is a process that results in a change in the behaviour, opinion or feelings of a human being as a result of what other people do, think or feel. The essence of social influence is of course not only exerting social influence, but also succumbing to it, which will be taken into account in the used model by means of appropriate parameters (intensity of persuasion and intensity of support). The Latané [15] theory rely on three experimentally proven [18–20] assumptions:

**social force principle** it says that social impact *I* (details are given in description of Eq (2)) on *i*-th actors is a function of the product of strength *S*, immediacy *J*, and the number of sources *N*; The strength of influence is the intensity, power or importance of the source of influence. This concept may reflect socio-economical status of the one that affects our opinion, his/her age, prestige or position in the society. The immediacy determines the relationship between the source and the goal of influence. This may mean closeness in the social relationship, lack of communication barriers and ease of communication among actors;
**psycho-social law** it states that each next actor *j* sharing the same opinion as actor *i* exerts the lower impact on the *i*-th actor;
**division of impact theory** it is based on the bystander effect and is observed as the errors of reacting to crisis events, along with an increase in the number of witnesses to this event.

Based on these assumptions Nowak et al. [21] proposed computerised model of opinion dynamics based on Latané [18–20] social impact theory (see Ref. [67] for review).

Every agent *i* is characterised by the following parameters:

**opinion *ξ*_*i*_** the current opinion supported by agent *i*,
**intensity of support *s*_*i*_** the strength of the agent *i* influence on other agents, which determines the ability of this agent to convince other agents not to change their opinion if this opinion is identical to his/her opinion (0 ≤ *s*_*i*_ ≤ 1),
**intensity of persuasion *p*_*i*_** the strength of agent *i* influence on agents, which determines the ability of this agent to convince other agents to accept his/her opinions (0 ≤ *p*_*i*_ ≤ 1).

Each agent is influenced by all other agents on the lattice. The strength of this influence decreases as the distance between agents increases. In the presented model a cellular automaton was used, which consists of a square grid of *L*^2^ cells, where exactly one agent is assigned to each cell. The distance *d*_*ij*_ between agents *i* and *j* is calculated as the Euclidean distance between cells.

To take into account the varied flow of information in the community, we use the *α* parameter, which was adapted to scale the distance function. Parameter *α* adjusts the influence of close and distant neighbours in the community. Small *α* values mean good communication between agents and good access to information, because it allows for an exchange of information with a large number of agents in the lattice. The larger values of *α*, weaker the communication among the groups of agents, weaker effective exchange of information and weaker access to information, because the exchange of information takes place only in the closest neighbourhood of actors, although we still keep long-range interactions among actors.

Here we are on a position to recapitulate the formal model composition as proposed in Ref. [16].

### Formal model description

Actors occupy the nodes of the square lattice with linear size *L*. Every actor 1 ≤ *i* ≤ *L*^2^ is characterised by his/her discrete opinion *ξ*_*i*_ ∈ {Ξ_1_, Ξ_2_, ⋯, Ξ_*K*_}, where *K* is the number of opinions available in the system. Additionally, we assign random real value *p*_*i*_ ∈ [0, 1] and *s*_*i*_ ∈ [0, 1] describing actor’s persuasiveness and his/her supportiveness, respectively.

The system evolution depends on the social temperature *T*. If *T* = 0, then a lack of noise is assumed, and the actor *i* adopts an opinion Ξ_*k*_ that has the most impact on it:

$$\begin{array}{c} \xi_{i}\left(t+1\right)=\Xi_{k}\Leftrightarrow I_{i,k}\left(t\right)=\max\left(I_{i,1}\left(t\right),I_{i,2}\left(t\right),\cdots ,I_{i,K}\left(t\right)\right), \end{array}$$ (1)

where *k* is the label of this opinion which believers exert the largest social impact on *i*-th actor and *I*_*i*,*k*_ are the social influence on actor *i* exerted by actors sharing opinion Ξ_*k*_.

The social impact on actor *i* from actors *j* sharing opinion of actor *i* (*ξ*_*j*_ = *ξ*_*i*_) is calculated as

$$\begin{array}{c} I_{i,k}\left(t\right)=4J_{s}(\sum_{j=1}^{N}\frac{q(s_{j})}{g(d_{i,j})}\delta \left(\Xi_{k},\xi_{j}\left(t\right)\right)\delta \left(\xi_{j}\left(t\right),\xi_{i}\left(t\right)\right)) \end{array}$$ (2a)

while *K* − 1 social impacts on actor *i* from all other actors having *K* − 1 different opinions (*ξ*_*j*_ ≠ *ξ*_*i*_) is given as

$$\begin{array}{c} I_{i,k}\left(t\right)=4J_{p}(\sum_{j=1}^{N}\frac{q(p_{j})}{g(d_{i,j})}\delta \left(\Xi_{k},\xi_{j}\left(t\right)\right)\left[1-\delta \left(\xi_{j}\left(t\right),\xi_{i}\left(t\right)\right)\right]), \end{array}$$ (2b)

where 1 ≤ *k* ≤ *K* enumerates the opinions and Kronecker’s delta *δ*(*x*, *y*) = 1 if *x* = *y* and zero otherwise [16].

As in Ref. [16] we assume identity function for scaling functions $J_{S}\left(x\right)\equiv x$, $J_{P}\left(x\right)\equiv x$, *q*(*x*)≡*x*. The distance scaling function should be an increasing function of its argument. Here, we assume the distance scaling function as

$$\begin{array}{c} g\left(x\right)=1+x^{\alpha }, \end{array}$$ (3)

what ensures non-zero values *g*(0) = 1 of denominator for self-supportiveness in Eq (2a).

The exponent *α* is an arbitrary quantity which characterise the long-range interaction among actors. For small values of *α* (for instance for *α* = 2) we assume good communication among actors, good access to information in the society and effective exchange of information. In contrary, for larger values of *α* (for instance for *α* = 6) discussion and information exchange takes place only in the actors’ nearest neighbourhood.

For *T* > 0, the larger the social temperature *T* (noise), the more often the opinions, that do not have the greatest impact are selected. As it was shown in Ref. [16] in the modelled system the phase transition occurs: below critical temperature *T* ≪ *T*_*c*_ the ordered phase is observed with domination of one of the available opinion, while for *T* ≫ *T*_*c*_ all opinions become equally supported by actors. Critical temperatures *T*_*c*_ (but for homogeneous society with ∀*i*: *s*_*i*_ = *p*_*i*_ = 0.5) are *T*_*c*_ = 6.1 and *T*_*c*_ = 4.7, for two and for three opinions, respectively [16]. In this article, simulations were carried out for *T*≤7 to take into account different levels of noise, reaching the critical level at which agents more often take random opinions than guided by the opinion of their neighbours.

For finite values of social temperature *T* > 0 we apply the Boltzmann choice

$$\begin{array}{c} p_{i,k}\left(t\right)=\exp(\frac{I_{i,k}\left(t\right)}{T}), \end{array}$$ (4a)

which yields probabilities

$$\begin{array}{c} P_{i,k}\left(t\right)=\frac{p_{i,k}\left(t\right)}{\sum_{j=1}^{K}p_{i,j}\left(t\right)} \end{array}$$ (4b)

of choosing by *i*-th actor in the next time step *k*-th opinion:

$$\begin{array}{c} \xi_{i}\left(t+1\right)=\Xi_{k},\;\text{with}\;\text{probability}\;P_{i,k}\left(t\right). \end{array}$$ (4c)

The form of dependence (4a) in statistics and economy is called logit function [11, 40].

Both, for *T* = 0 and *T* > 0 the calculated social impacts *I*_*i*,*k*_(*t*) influence the *i*-th actor opinion *ξ*_*i*_(*t* + 1) at the subsequent time step. Newly evaluated opinions are applied synchronously to all actors. The simulations takes one thousand time steps which ensures reaching a plateau in time evolution of several observables.

The simulations are carried out on square lattice of linear size *L* = 41 with open boundary conditions. To check the system response to its size varying, also simulations for *L* = 21 and *L* = 61 were carried out. We assume random values of supportiveness *s*_*i*_ and persuasiveness *p*_*i*_ for all actors. The studies for homogeneous society, i.e. with ∀*i*: *p*_*i*_ = *s*_*i*_ = 0.5 were carried out in Ref. [16]. The results are averaged over one hundred independent simulations with various initial distribution of opinions and actors persuasiveness and actors supportiveness.

The example of social impact calculations for a small system (with nine actors and three opinions) is given in Supporting information. The model implementation in Fortran95 [68] is attached as Listings 1 and 2 in Supporting information.

## Results

### Spatial distribution of opinions

We start presentation of our results by showing the spatial distribution of opinions for *K* = 2, 3 (various numbers of opinions available in the system), for *α* = 2, 3, 6 (various levels of flow of information), for *T* = 0, 1, 3, 5 (various levels of noise).

#### Two opinions, *K* = 2

**Fig 1:**
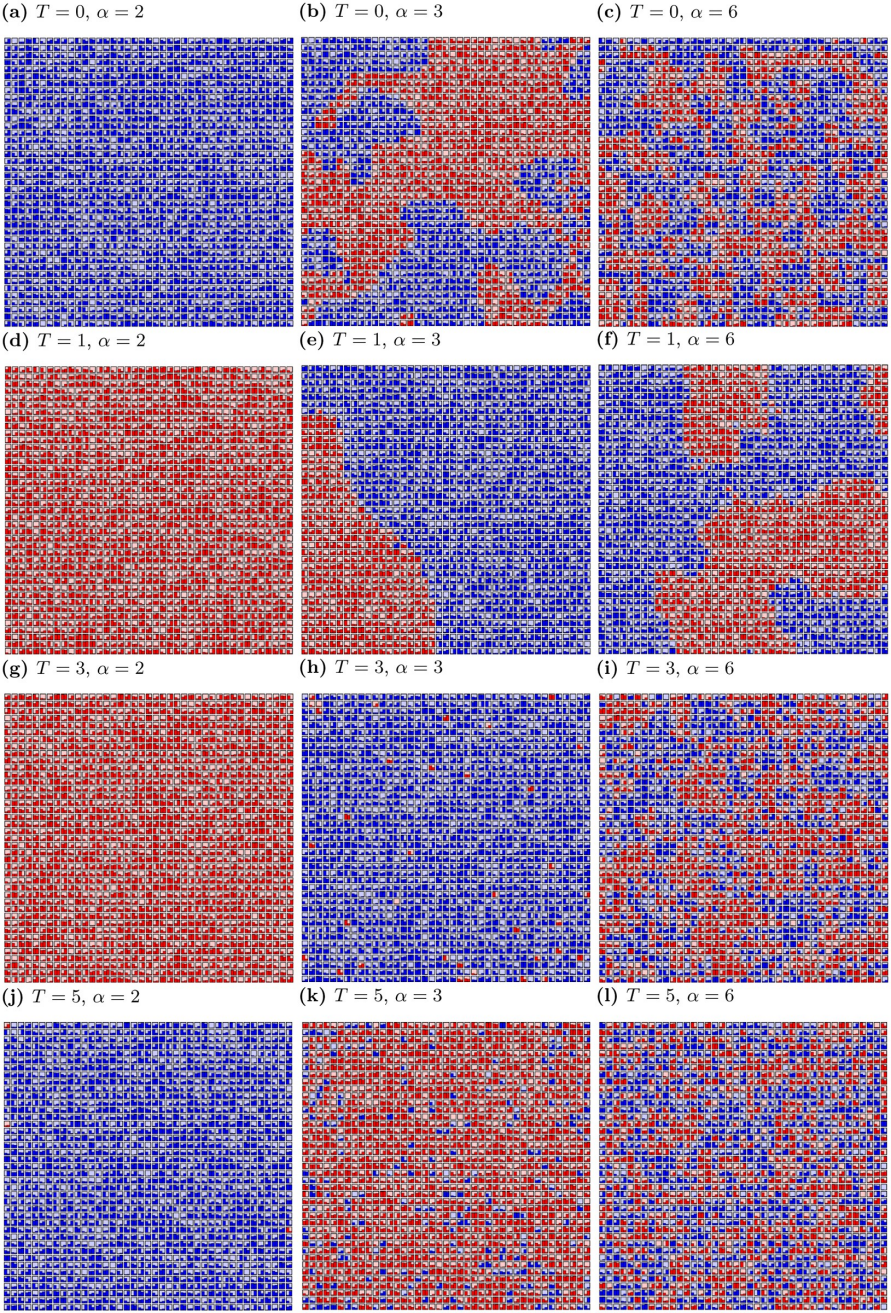
Spatial distribution of opinions *ξ* for various values of social temperature *T* and exponent *α* after *t*_max_ = 10^3^ time steps. *L*^2^ = 1681, *K* = 2. Pictures are produced with application [69].

In Fig 1 the simulation results for *K* = 2, *α* = 2, 3, 6 and *T* = 0, 1, 3, 5 are presented.

Both, *α* (information flow) and *T* (noise) influence opinion formation and the spatial distribution of opinions. For *α* = 2 the consensus takes place (all actors adopt one of two opinions, except of few actors for large *T* values). Interesting phenomena for *α* = 3 are observed, where the frozen initial system goes into a polarised phase of two large clusters for *T* = 1 and into a consensus phase for *T* = 3 (one cluster with single actors with a different opinion) before disordering for *T* = 5. For *α* = 6 many clusters are visible, although the introduction of noise (*T* = 1) results in a more ordered system than for other *T* values. To sum up, the increase of *α* and *T* generally causes greater disorder in the system (many clusters with both opinions), but for some values of these parameters their increase leads to order (consensus)—see Fig 1h.

In general, we can observe four main types of structures in the formation of opinions for *K* = 2:

- formation of single cluster, when all agents adopt one opinion and consensus takes place (Fig 1a, 1d, 1g and 1j),
- the majority of agents with the same opinion and single agents with opposing opinions scattered across the lattice (Fig 1h),
- formation of several large clusters of agents with different opinions—polarisation of the group opinion (Fig 1e),
- formation of plenty small clusters with both opinions (e.g. Fig 1b and 1f).

#### Three opinions, *K* = 3

**Fig 2:**
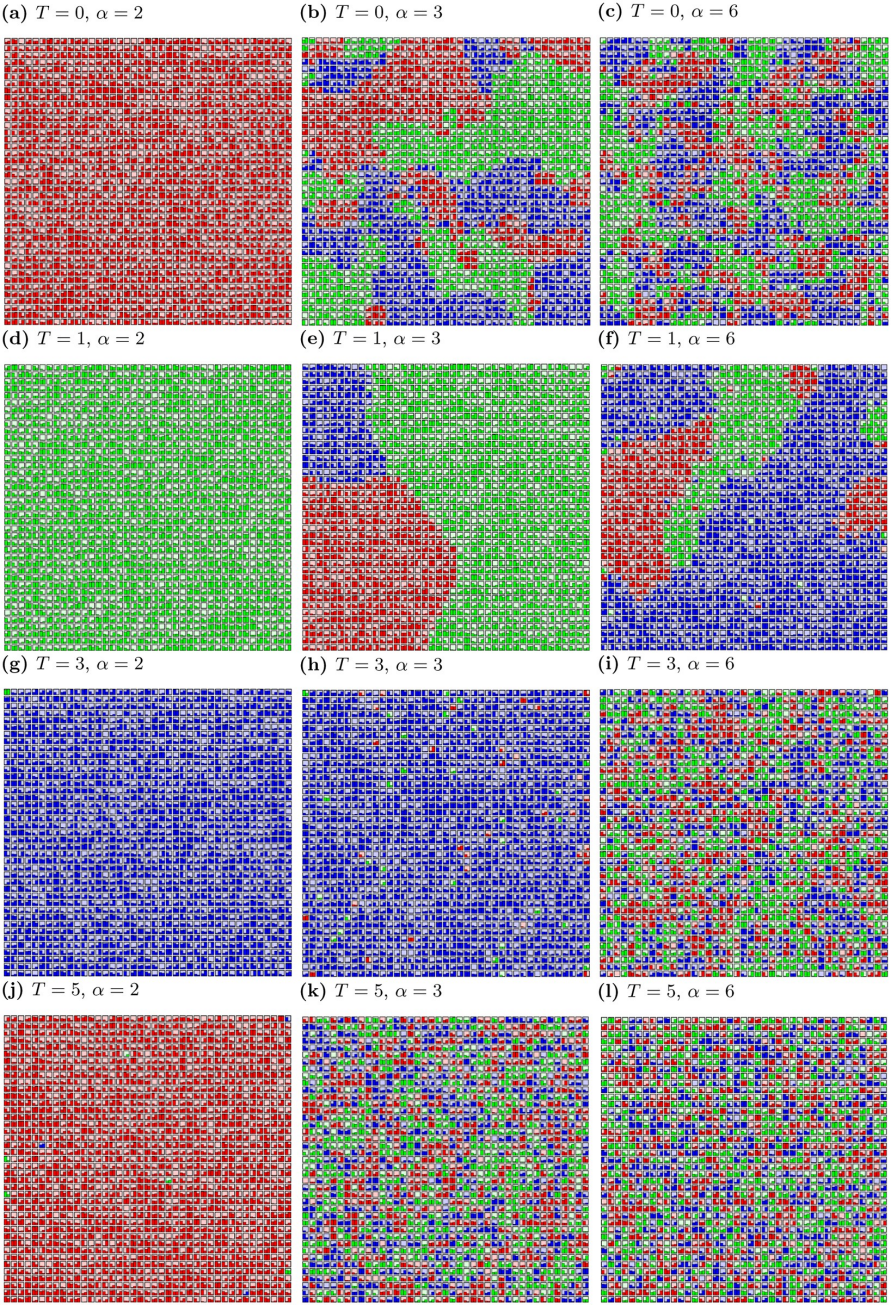
Spatial distribution of opinions *ξ* for various values of social temperature *T* and exponent *α* after *t*_max_ = 10^3^ time steps. *L*^2^ = 1681, *K* = 3. Pictures are produced with application [69].

The simulation results for three opinions among actors (where *K* = 3, *α* = 2, 3, 6 and *T* = 0, 1, 3, 5) are presented in Fig 2.

Similarly to *K* = 2, the formation of opinions (the formation of clusters of opinion) depends on the level of noise and the effective range of interactions among actors. For *α* = 2, one cluster is formed—consensus take place. For *K* = 3 the consensus among actors with three different opinions is also possible for *α* = 3. In other cases, many clusters with three opinions or disordered system state are visible.

In general, we can observe four types of structures in the formation of opinions for *K* = 3, after thousand time steps:

- formation of single cluster, when all agents adopt one opinion and consensus takes place (Fig 2a, 2d, 2g and 2j),
- the majority of agents with the same opinion and single agents with opposing opinions scattered across the lattice (Fig 2h),
- formation of clusters with all possible opinions—polarisation of the group opinion (Fig 2e),
- formation of plenty small clusters with all opinions (e.g. Fig 2b, 2c and 2f).

### Clustering of opinions

**Fig 3:**
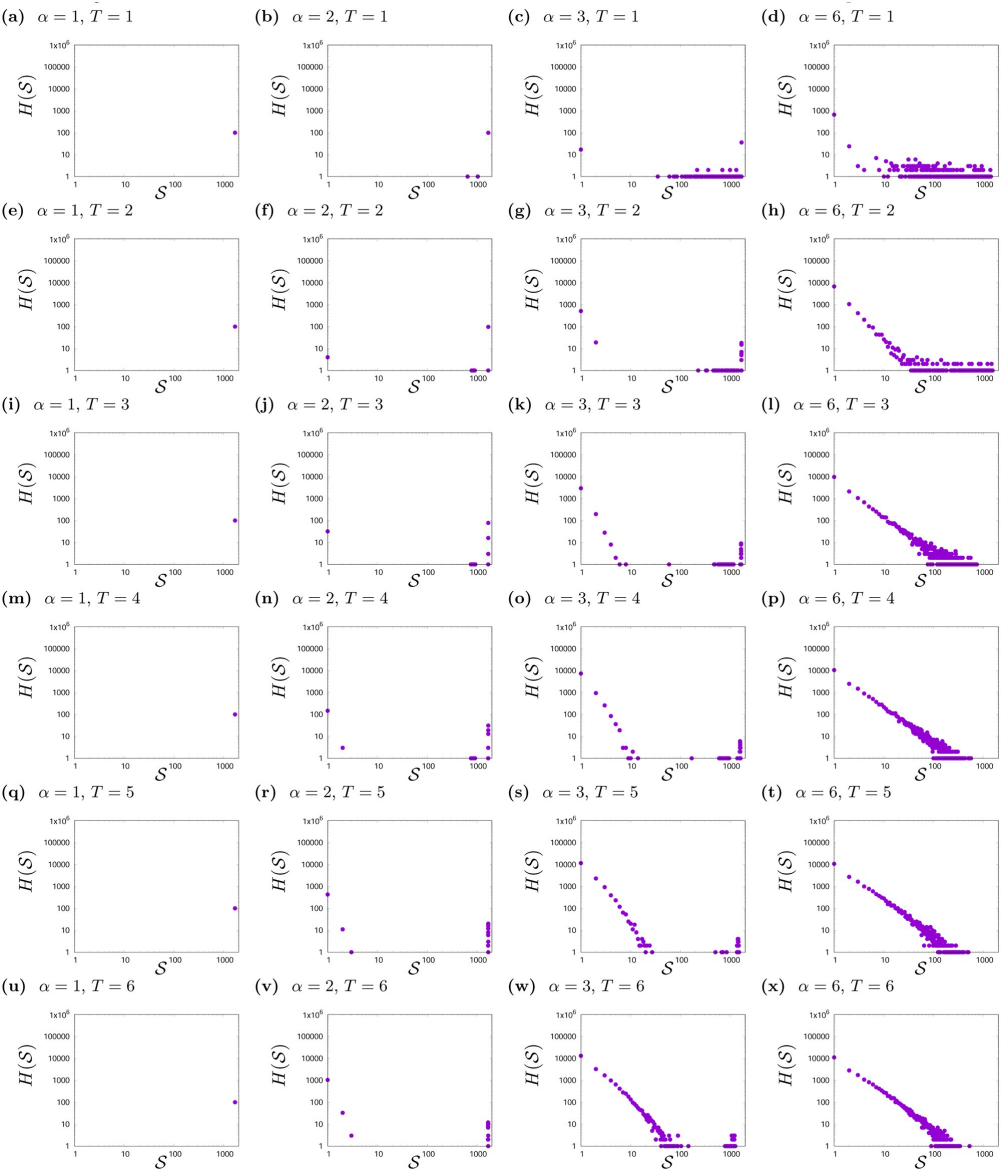
Histograms H(S) of cluster sizes S for various values of social temperature *T* and exponent *α*. *L* = 41, *K* = 2. The results are gathered from hundred simulations with different initial conditions after *t*_max_ = 1000 time steps.

**Fig 4:**
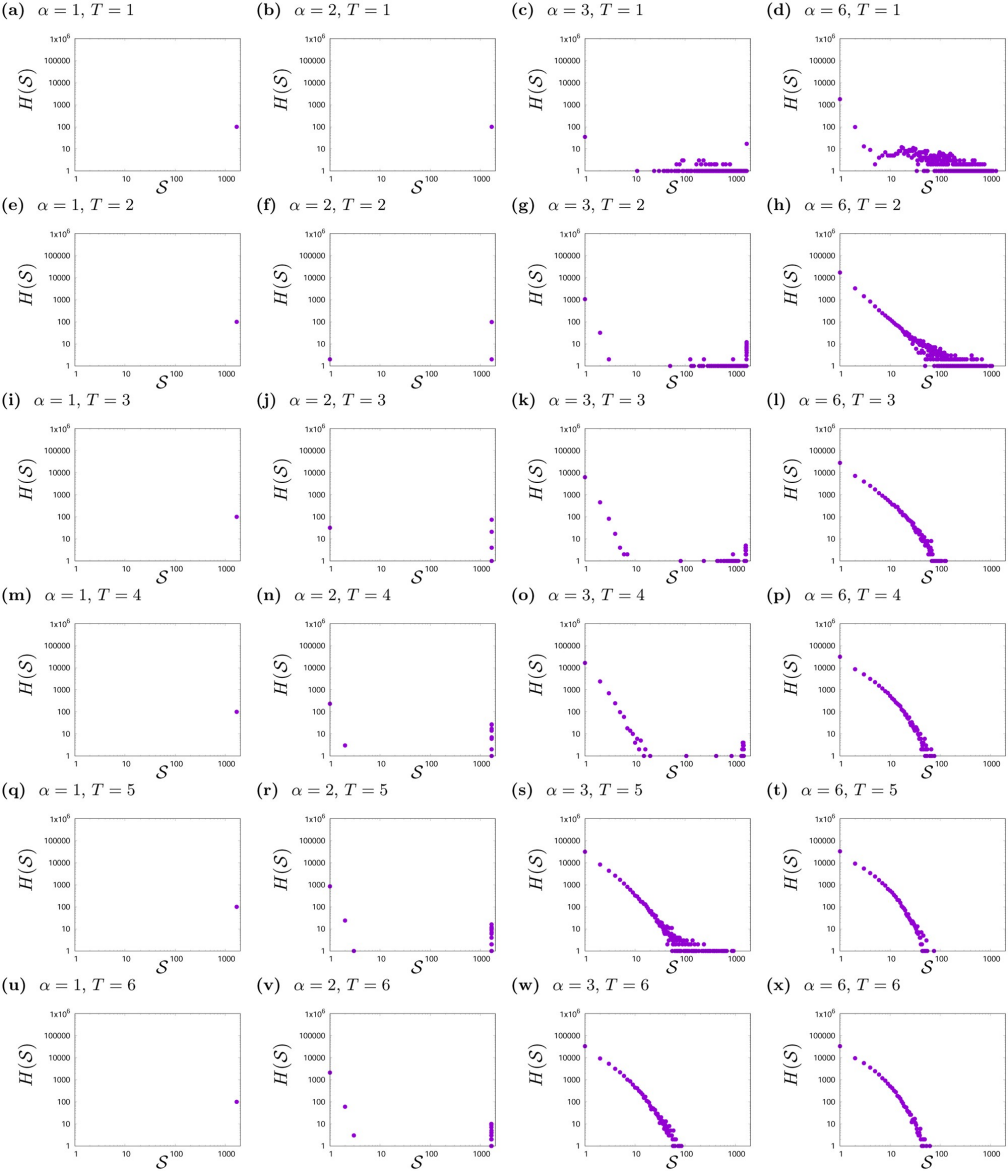
Histogram H(S) of cluster sizes S for various values of social temperature *T* and exponent *α*. *L* = 41, *K* = 3. The results are gathered from hundred simulations with different initial conditions after *t*_max_ = 1000 time steps.

**Fig 5:**
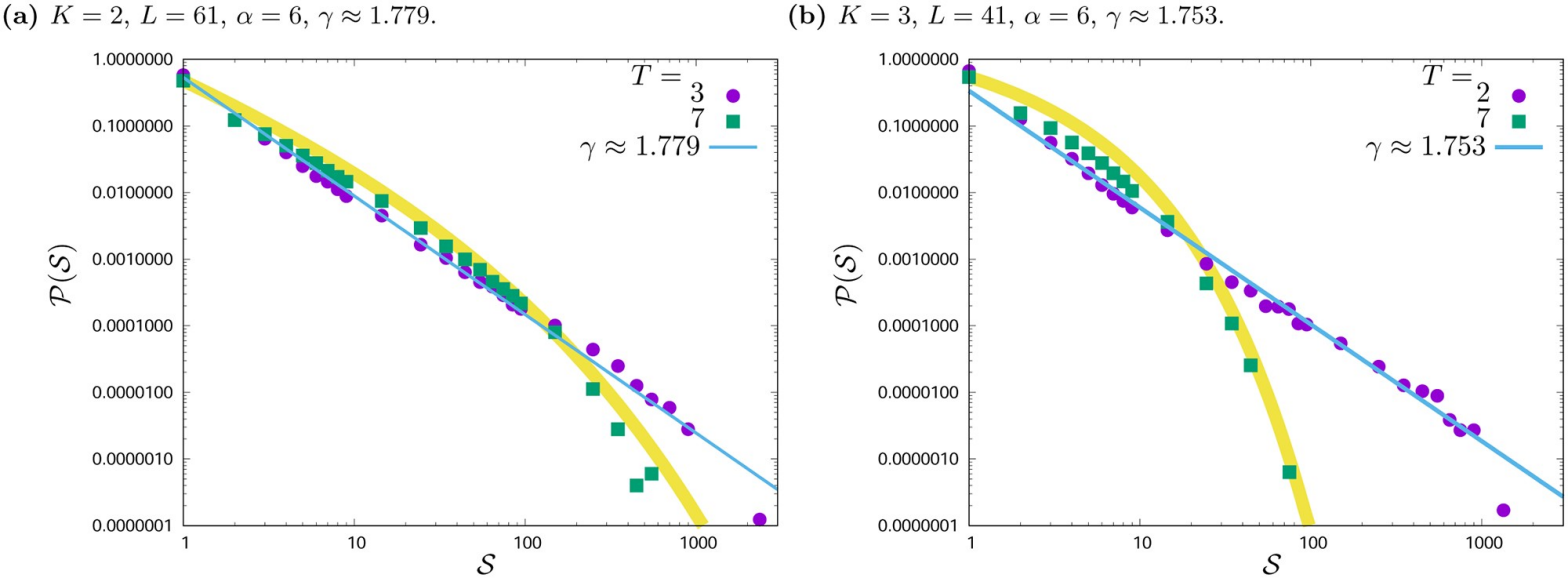
Probability distribution function $P\left(S\right)=H\left(S\right)/\sum_{S=1}^{L^{2}}H\left(S\right)$ of clusters sizes S for *L* = 61 (*K* = 2), *L* = 41 (*K* = 3), *α* = 6. Data are binned in intervals of length ΔS=1, 10, 100 for 1 ≤ *s* ≤ 9, 10 ≤ *s* ≤ 99, 100 ≤ *s* ≤ 999, respectively. For *K* = 2 additional simulations for *L* = 61 were carried out and their results are presented in Fig 5a. The fits for *K* = 2, *T* = 3 and *K* = 3, *T* = 2 follow scale-free distribution $P\left(S\right)\propto S^{-\gamma }$. The thick yellow line is a guide for eye for P(S) dependencies for *T* = 7.

In Figs 1 and 2—discussed in the previous section—different phases of the system depending on the parameters *α* and *T* were presented. In order to get a better look at influence of *T* and *α* on system behaviour, we analysed the histograms H(S) of cluster sizes S after thousand steps of simulation gathered from hundred simulations (see Figs 3, 4 and 5).

We apply the Hoshen–Kopelman algorithm [70] for clusters detection. In Hoshen–Kopelman algorithm each actor is labelled in such way, that actors with the same opinions and in the same cluster have identical labels. The algorithm allows for cluster detection in multi-dimensional space and for complex neighbourhoods [71–75], here however, we assume the simplest case, i.e. square lattice with von Neumann neighbourhood.

To better explain the phenomena observed in Figs 3 and 4, the following parameters describing the number and size of clusters were selected:

- average largest cluster size $\langle S_{\max}\rangle$,
- average cluster number 〈*n*_*c*_〉,
- average number of small clusters (i.e. clusters with sizes less or equal to five cells) 〈*n*_*s*_〉.

The example of clusters

**Fig 6:**
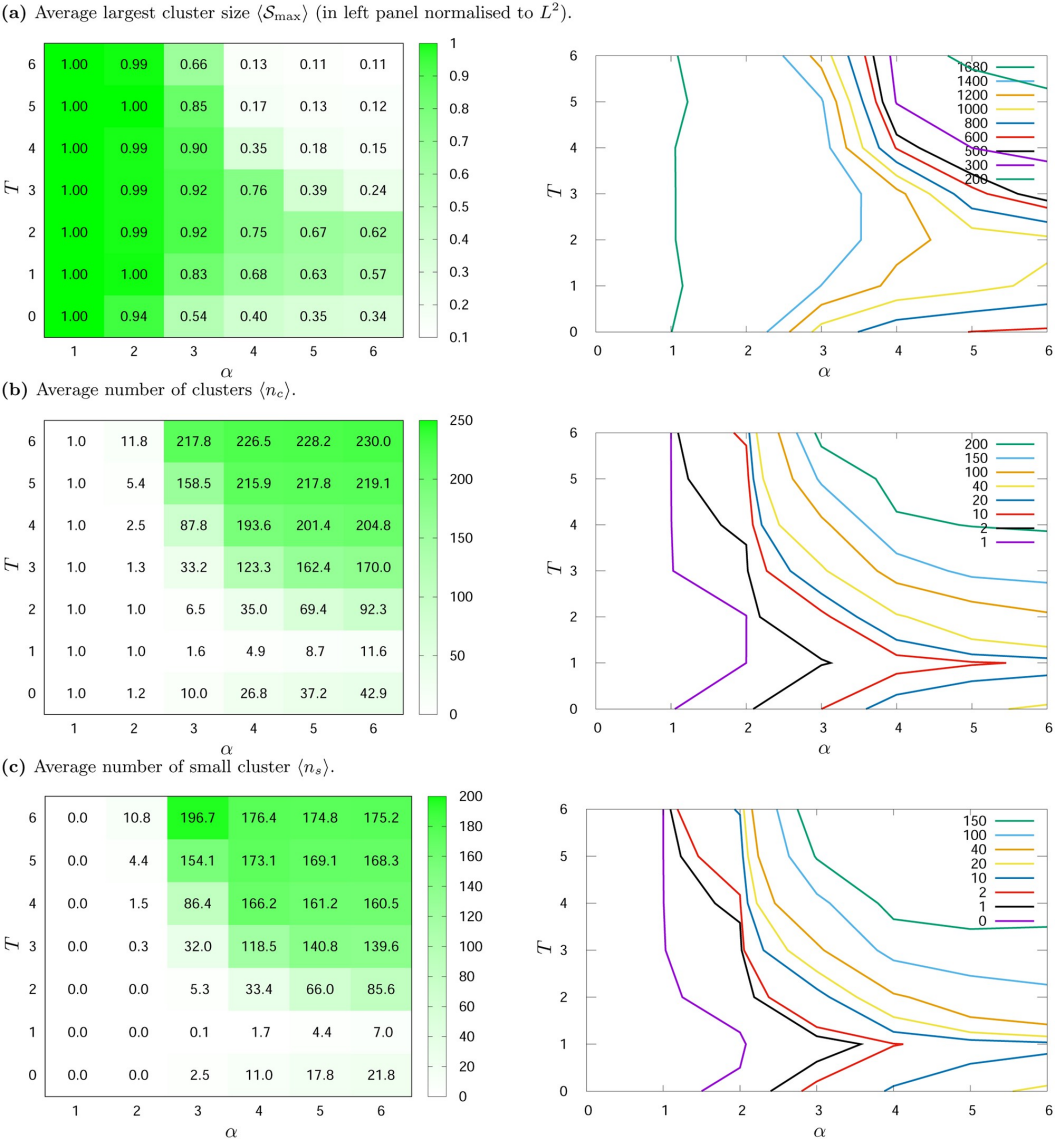
Average (a) largest cluster size $\langle S_{\max}\rangle$, (b) number of clusters 〈*n*_*c*_〉 and (c) average number of small clusters 〈*n*_*s*_〉 for various values of noise level *T* and exponent *α*. *L*^2^ = 1681, *K* = 2. The results are averaged over hundred simulations with different initial conditions and measured after *t*_max_ = 10^3^ time steps.

**Fig 7:**
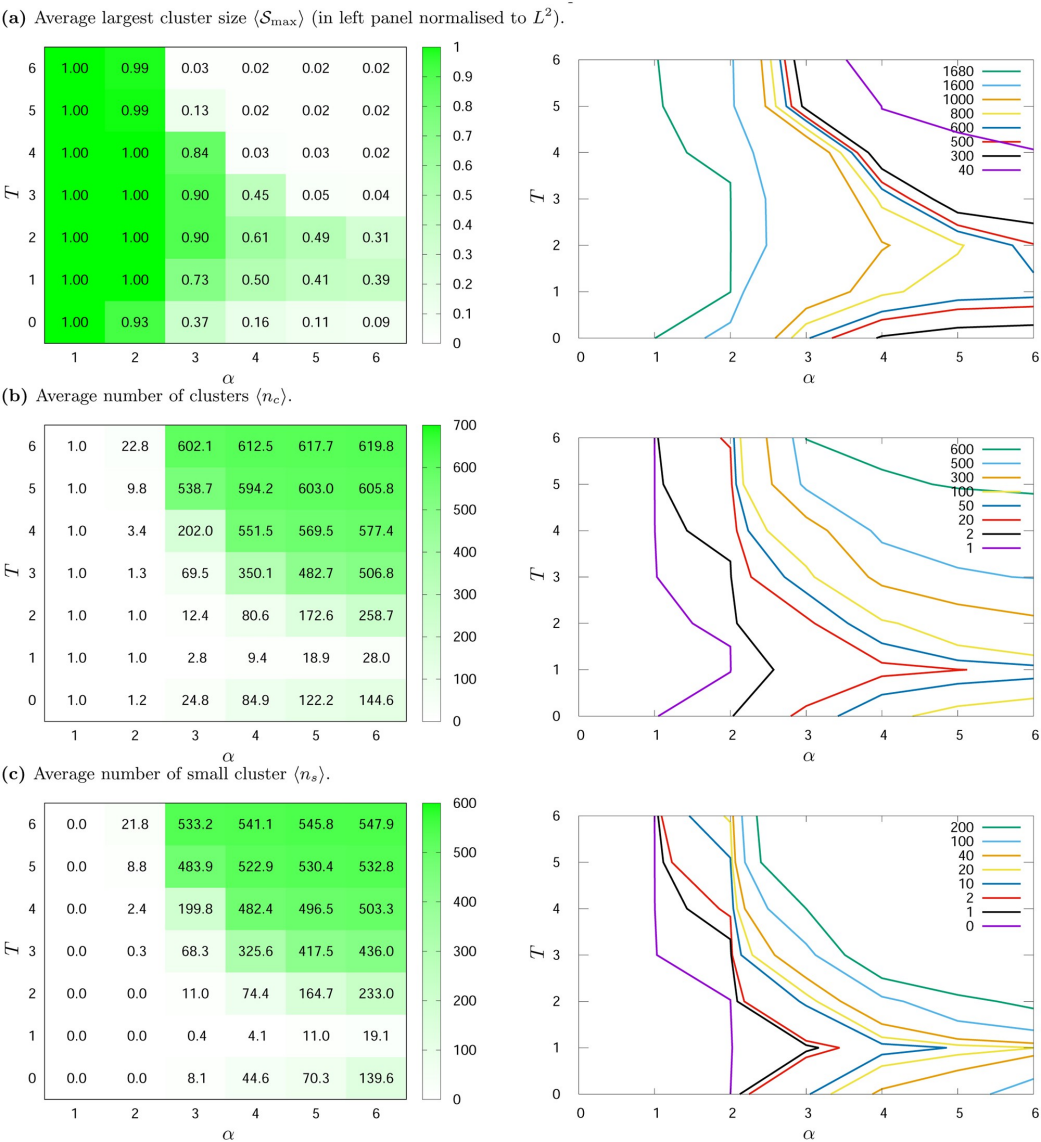
Average (a) largest cluster size $\langle S_{\max}\rangle$, (b) number of clusters 〈*n*_*c*_〉 and (c) average number of small clusters 〈*n*_*s*_〉 for various values of noise level *T* and exponent *α*. *L*^2^ = 1681, *K* = 3. The results are averaged over hundred simulations with different initial conditions and measured after *t*_max_ = 10^3^ time steps.

- size distribution counting (for preparation histograms H(S) for Figs 3 and 4),
- average largest value $\langle S_{\max}\rangle$ (for Figs 6a and 7a),
- and average numbers 〈*n*_*c*_〉 (for Figs 6b and 7b) and 〈*n*_*s*_〉 (for Figs 6c and 7c)

are provided in Supporting information.

On the left panels of Fig 6 (for *K* = 2) and Fig 7 (for *K* = 3) the ‘heat maps’ and numerical values of $\langle S_{\max}\rangle$, 〈*n*_*c*_〉 and 〈*n*_*s*_〉 for different values of *α* and *T* are presented. The supplementary ‘contour maps’, presenting the same data but allowing for better visualisation of non-monotonous character of these data, are included on the right panels of these figures. On left panels average values of the largest cluster sizes $\langle S_{\max}\rangle$ are expressed as a fraction of the number of sites (*L*^2^). The results were averaged over hundred simulations and gathered after *t*_max_ = 1000 time steps.

As can be seen in Figs 3 and 4, the shapes of histograms are very similar to each other for *K* = 2 (Fig 3) and *K* = 3 (Fig 4). In both cases, there are clear differences in the shape of histograms due to values of *α* (information flow, effective range of interaction among actors):

- For *α* = 1 and *α* = 2—where the effective range of interaction is the largest—the unanimity of opinions is achieved (in the case of *α* = 2, single agents and rarely small clusters with opposite opinions may appear).
- The most interesting are the histograms for *α* = 3 (see figures forming columns from Fig 3c to 3w and from Fig 4c to 4w), because the difference in the shape of histograms due to noise (*T*) is also visible. For *α* = 3, various phases in the system behaviour are observed. The system from the disordered state, with the growth of *T* is increasingly ordered. As *T* increases, more and more clusters appear close to the maximum cluster size in this lattice and more and more small clusters consisting of single agents.
- For *α* = 6, histograms (see figures forming columns from Fig 3d to 3x and from Fig 4d to 4x) indicate the phase of system disorder (a large number of clusters), apart from slight orderliness for *T* = 1 (see Figs 3d and 4d). Moreover, in this phase of the system disorder, we observe a change in the shape of the histograms H(S) for both *K* = 2 and *K* = 3. For two opinions, this change is visible between *T* = 3 (Fig 3l) and *T* = 4 (Fig 3p), and for three opinions—between *T* = 2 (Fig 4h) and *T* = 3 (Fig 4l). Such effect (not shown) for *K* = 2 also occurs for *α* = 4 between *T* = 4 and *T* = 5 and for *α* = 5 between *T* = 3 and *T* = 4. For system with three available opinions—and in disordered phase—the change of histograms shapes occurs when noise level changes from *T* = 2 to *T* = 3 (for *α* = 5); from *T* = 3 to *T* = 4 (for *α* = 4) and from *T* = 5 to *T* = 6 (for *α* = 3). In order to better visualise this effect we compose Fig 5, where probability distribution function $P\left(S\right)=H\left(S\right)/\sum_{S=1}^{L^{2}}H\left(S\right)$ are presented. The data for *K* = 2, *α* = 6, *T* = 3; *K* = 2, *α* = 6, *T* = 7; *K* = 3, *α* = 6, *T* = 2 and *K* = 3, *α* = 6, *T* = 7 are additionally aggregated from intervals of length ΔS=1, 10, 100 for 1≤S≤9, 10≤S≤99, 100≤S≤999, respectively. For *K* = 2, *T* = 3 (Figs 3l and 5a) and *K* = 3, *T* = 2 (Figs 4h and 5b) the binned data follow a scale-free distribution $P\left(S\right)\propto S^{-\gamma }$, with *γ* ≈ 1.76 independently on values of *K*. For *K* = 2, *T* = 4 (Figs 3p and 5a) and *K* = 3, *T* = 3 (Figs 4l and 5b) the deviations from the scale-free distribution are observed with evident shrinking of range of clusters sizes S. As this deviation for *K* = 2 and *L* = 41 is not too strong we construct P(S) for *K* = 2 basing on lattice with *L* = 61, where this shrinking effect is slightly enhanced in comparison with *L* = 41.

In Figs 3 and 4, we can also notice that for *α* > 2, the greater the randomness in adopting opinions by agents (greater *T*), the more single clusters containing one agent and several agents appear.

#### Two opinions, *K* = 2

As can be seen in Fig 6a, the average size of the maximum cluster $\langle S_{\max}\rangle$ decreases with *α* for fixed *T* values. The appearance of noise in the system (*T* = 1) slightly organises the system in relation to the noiseless situation with *T* = 0 (which is particularly visible for *α* > 2, see Fig 6a). Indeed, like in earlier studies [64, 66], small level of noise brought more order to the system. In addition, the introduction of noise (*T*) in the adoption of opinions causes an increase in $\langle S_{\max}\rangle$, and then its decrease, which is especially visible for *α* > 2 (this inflection point is nearly *T* = 2). Particularly interesting are the large values of $\langle S_{\max}\rangle$ for *α* = 3 and *T* = 2, 3, 4. This issue will be discussed below.

Fig 6b shows the simulation results of the average number of clusters 〈*n*_*c*_〉. As can be seen, for fixed noise level *T*, the average number of clusters 〈*n*_*c*_〉 increases with *α*. Comparing the results for all *α* values and for the noiseless system (*T* = 0) with the results for small noise level (*T* = 1), it can be seen that the introduction of noise results in system ordering. This figure also shows that for *α* > 2 the average number of clusters increases with *T* for all *T* > 1.

The average number of small clusters 〈*n*_*s*_〉 behaves non-monotonically with *α* and *T* (see Fig 6c). For *α* = 1 there are no small clusters, and for *α* = 2 there are few of them. The increase in the number of small clusters due to *T* and *α* is well visible for *α* > 2, i.e. when the range of interaction between agents is small.

Considering all results presented in Fig 6 we can notice, that:

- For *α* ≤ 2 we observe the system phase in which there is a consensus (one large cluster representing actors sharing one of the available opinions). The average size of the largest cluster is equal to the size of the lattice. For *α* = 2 and *T* > 2, the average number of clusters is larger than one, but these higher 〈*n*_*c*_〉 values only mean the appearance of clusters consisting of single agents, as |〈*n*_*c*_〉 − 〈*n*_*s*_〉| ≈ 1, which is shown in Fig 6b and 6c and also visible in Fig 3j, 3n, and 3r.
- An interesting phenomenon can be observed for *α* = 3, where the frozen noiseless system goes into a more ordered phase for *T* = 1 and *T* = 2, and into a consensus phase for *T* = 3 before disordering at *T* = 5 (see Fig 6a, 6b and 6c for *α* = 3). Polarisation of opinion is observed for *T* = 1 (about 60% of simulations end with polarisation and about 40% of them end with consensus). The largest cluster contains (in average) 83% of all agents on the lattice (see Fig 6a—left panel). The average number of clusters is very small and it equals 1.6 (see Fig 6b—left panel), and the average number of small clusters is 0.1 (see Fig 6c—left panel), which means that the simulations mostly end in a state containing two clusters, one of which is definitely larger than the other. This is also visible in Figs 1e and 3c. For *T* = 2, more and more system ordering is observed (more and more simulations end with unanimity).
  The second phase in the case of *α* = 3 takes place for *T* = 3. Despite the high values of noise *T*, the size of the maximum cluster $\langle S_{\max}\rangle$ is still very large and contains about 90% of all actors (Fig 6a), and the average number of clusters is definitely larger than for *T* ≤ 2 (see Fig 6b). An increase in noise level surprisingly causes a kind of order. In this case, one of the opinions dominates, but representatives of the opposite opinion appear in the form of small individual clusters. The average number of small clusters 〈*n*_*s*_〉 is 32 for *T* = 3. This number when compared to the average number of clusters 〈*n*_*c*_〉 in Fig 6b (33.2 for *T* = 3) indicates the emergence of one large cluster and individual small clusters (the average number of small clusters 〈*n*_*s*_〉 differs by approximately one from the average number of clusters 〈*n*_*c*_〉). This means, that system evolution toward unanimity of opinion dominates system dynamics for *T* = 3.
  For *T* = 4, the size of the largest cluster $\langle S_{\max}\rangle$ is still very large (and reaches ca. 90% of all actors), but although 〈*n*_*c*_〉 differs from 〈*n*_*s*_〉 by about one, both the number of clusters and the number of small clusters are definitely larger than in the case of *T* = 3 (Fig 6a, 6b and 6c). So, for *T* = 4, the system goes into a disordered phase.
  In the case of *T* ≥ 5, the system enters a phase of disorder, because there are definitely more clusters with opposite opinions and they have a larger size than for *T* = 3 and *T* = 4, which is also visible in Figs 3s and 1k.
- For *α* = 4 and *α* = 5, when the noise level goes to *T* = 2, a slight increase of noise induces more order. This can be seen in the average size of the largest cluster ($\langle S_{\max}\rangle$ for *T* = 2 is larger than for *T* = 1—see Fig 6a) and by comparing the average number of clusters 〈*n*_*c*_〉 with the average number of small clusters 〈*n*_*s*_〉 (the difference is smaller than for *T* = 1)—Fig 6b and 6c. When the interaction effectively takes place only among the nearest neighbours (for *α* = 6), this effect vanishes.
  As it was mentioned earlier, for *α* = 4, 5 and 6 the change in the histogram shape was observed. This phenomenon is weaker in the case of *K* = 2 than *K* = 3 (see Fig 5) and in Fig 6a, 6b and 6c (for *α* = 4 and *T* = 5; for *α* = 5, 6 and *T* = 4) it is not clearly visible, although a decrease in the average largest cluster size $\langle S_{\max}\rangle$, and an increase in the average number of clusters 〈*n*_*c*_〉 and the average number of small clusters 〈*n*_*s*_〉, is observed.

To check the system response to its size varying for *K* = 2, simulations for *L* = 21 and *L* = 61 were also carried out. The simulations for the smaller and larger lattice of agents showed results consistent with the presented for lattices with size *L* = 41. Slight differences were observed for *α* = 3, where for *T* = 1 and *T* = 2 after 1000 steps of simulation, in the case of a smaller lattice, consensus is more often observed, and for a larger lattice less often. Moreover, for *α* = 6 the larger lattice size *L* = 61 slightly enhances difference between the shapes of histograms H(S) for *T* = 3 and *T* = 7. Thus the data presented in Fig 5a comes from simulations for *L* = 61.

To sum up, three main types of structures in the formation of opinions for *K* = 2 are observed:

- formation of single cluster, when all agents adopt one opinion and consensus takes place (for *α* ≤ 2, *T* ≥ 1 and *α* = 3, *T* = 3),
- greater orderliness—polarisation of opinions in clusters (*α* = 3, *T* = 1, 2),
- formation of plenty small clusters with both opinions—disorder (for other values of *α* and *T*). For *α* = 4, 5 and 6 the shape of histograms *H*(*S*) in disordered phase changes from scale-free behaviour to yet another distribution. This phenomenon is observed in the case of *α* = 4 when noise level changes from *T* = 4 to *T* > 4, and in the case of *α* = 5 and 6 when noise level changes from *T* = 3 to *T* > 3. This effect is particularly well visible for *α* = 6 in high noise level limit (here, *T* = 7, see Fig 5a).

#### Three opinions, *K* = 3

In Fig 7 the simulation results for *K* = 3 are presented. The average size of the largest cluster $\langle S_{\max}\rangle$ (Fig 7a) decreases with *α* for fixed values of the noise level *T*, as it was observed for *K* = 2. For *α* = 1 and *α* = 2, $\langle S_{\max}\rangle$ is equal to the number of all actors on the lattice. For *α* > 2, the average size of the largest cluster $\langle S_{\max}\rangle$ increases up to a certain value of *T*, and then decreases. This inflection point is nearly *T* = 2.

In the case of the average number of clusters 〈*n*_*c*_〉—which is presented in Fig 7b—this number is the smallest for *T* = 1, i.e. as soon as noise is introduced to the system. Similarly to the case of *K* = 2, for fixed noise level *T*, the average number of clusters 〈*n*_*c*_〉 increases with *α*. Fig 7b also shows, that for *α* > 2 the average number of clusters 〈*n*_*c*_〉 increases with *T* for all *T* > 1.

The average number of small clusters 〈*n*_*s*_〉, similarly to the case of *K* = 2, behaves non-monotonically with *T* and *α* (see Fig 7c). For *α* = 1 there are no small clusters, and for *α* = 2 they appear only for *T* > 3. The increase in the number of small clusters 〈*n*_*s*_〉 with increase of *T* and *α* is clearly visible for *α* > 2.

As the simulation results suggest—also in the case of *K* = 3 opinions available in the system—various phases in the system behaviour can be observed. These phases are induced by interplay between noise level *T* and effective range of interaction among actors *α*. First of all, for *α* = 1 and all *T* values, a large cluster with one opinion is formed (consensus takes place). In this case, $\langle S_{\max}\rangle$ are equal to the number of all agents on the lattice, and the average number of clusters 〈*n*_*c*_〉 is one (see Fig 7a and 7b—left panels). Single cluster is also created in the case of *α* = 2. As can be seen in Fig 7a and 7b the average largest cluster size is close to the system size $\langle S_{\max}\rangle \approx L^{2}$, and the average number of clusters is close to one 〈*n*_*c*_〉≈1. The exceptions occur for large values of the noise level *T* ≥ 4, but this is associated with the appearance of single small and short-living clusters, which is typical for high randomness in actors behaviour. In this situation the average number of small clusters differs by approximately one from the average number of clusters |〈*n*_*c*_〉 − 〈*n*_*s*_〉| ≤ 1. These results are confirmed by the data in Fig 7b and 7c.

As in the case of two opinions (*K* = 2), interesting phenomena are visible for *α* = 3. For *T* = 1, polarisation of opinions is observed. In 80% of cases of the final system state two or three clusters of opinions are created, one of which is larger than the others ($S_{\max}/L^{2}=0.73$, see Fig 2a). For *T* = 2, as in the case of two opinions, more ordering is observed than for *T* = 1. Consequently, by increasing noise level, for *T* = 3 a consensus takes place (one large cluster and small clusters with only single actor). The average size of the largest cluster is still very large and contains about 90% of all actors in the lattice. Although the average number of clusters 〈*n*_*c*_〉 is quite large, when compared with the average number of small clusters 〈*n*_*s*_〉 it indicates the existence of one large cluster and single small clusters (the average number of clusters differs by approximately one from the average number of small clusters |〈*n*_*c*_〉 − 〈*n*_*s*_〉| ≈ 1—see Fig 7b and 7c). For *α* = 3 and *T* = 4, despite the still high $\langle S_{\max}\rangle$, the number of small clusters increases, which leads to a disorder phase for *T* = 5. The difference between average number of clusters 〈*n*_*c*_〉 and average number of small clusters 〈*n*_*s*_〉 is definitely greater than one cluster |〈*n*_*c*_〉 − 〈*n*_*s*_〉| ≫ 1 (cf. Fig 7a and 7b).

For *α* > 3 and all *T* values, system disorder (many clusters of all opinions) is visible—Figs 2 and 4, with the exception of *α* = 4 and *α* = 5, when a slight increase of noise (*T* = 2) does induce more order than for *T* = 1 (see Figs 7 and 4). In addition, in Fig 7a, 7b and 7c, the noise-induced transition that was visible in Fig 4 is also observed. For *α* = 3 and *T* = 6; *α* = 4 and *T* = 4; and for *α* = 5, 6 and *T* = 3 a sudden decrease in the average largest cluster size $\langle S_{\max}\rangle$, together with a sudden increase in the average number of both clusters 〈*n*_*c*_〉 and the average number of small clusters 〈*n*_*s*_〉, can be observed (this effect for *K* = 3 is stronger than for *K* = 2, even for larger lattice size as presented in Fig 5).

As for *K* = 2, in the case of three opinions, simulations for *L* = 21 and *L* = 61 were also carried out. The simulations for the smaller and larger lattice of agents showed similar results to the presented for lattice with size *L* = 41. Slight differences were observed for *α* = 3, and *T* = 1 and *T* = 2 after 1000 steps of simulation, where ordering in clusters is more visible in small networks than in large ones.

To sum up, we can notice three main phases in the behaviour of the model for *K* = 3:

- formation of single cluster, when all agents adopt one opinion and consensus takes place (for *α* ≤ 2, *T* ≥ 1 and *α* = 3, *T* = 3),
- greater orderliness—polarisation of opinions in clusters (sometimes one cluster or ordering opinions in two or three clusters, where two or three opinions occur (*α* = 3, *T* = 1, 2),
- formation of plenty clusters with all three opinions—disorder (for other values of *α* and *T*). For *α* ≥ 3 the shape of histograms H(S) in disordered phase changes abruptly from scale-free behaviour to yet another distribution. This phenomenon is observed in the case of *α* = 3 when noise level changes from *T* = 5 to *T* > 5, for *α* = 4: from *T* = 3 to *T* > 3, and in the case of *α* = 5 and 6: from *T* = 2 to *T* > 2. This effect is particularly well visible in high noise level limit (here, *T* = 7, see Fig 5b).

## Summary and conclusions

In this paper, we are interested in how opinions are formed and how they spread in the community. We were investigating how flow of information in the community and randomness of human behaviour influence formation of opinions, its spreading and its polarisation. The community was presented as a square lattice of linear size *L* with open boundary conditions, which is fully filled by actors.

The flow of information was controlled by the parameter *α*. This parameter reflects the effective impact of the neighbourhood on the opinion of the actors. In case of low values of this parameter, actors shape their opinion basing on a large number of actors (including distant neighbours). In our research, we also take into account the randomness in adopting opinions, which is expressed in the noise parameter *T*. The larger *T*, the more often actors adopt opinions which have no greatest impact on them.

Each actor in our model is characterised, in addition to the opinion, by two parameters. They are the intensity of persuasion (*p*_*i*_) and the intensity of support (*s*_*i*_). The higher the value of persuasiveness *p*_*i*_, the actor more easier convincing other actors to accept his/her opinion. With bigger (*s*_*i*_), the agent convinces more strongly other actors. These parameters therefore determine the effectiveness of which an individual may interact with or influence other individuals by changing or confirming their opinions. In all performed simulations, we adopted random values of (*p*_*i*_) and (*s*_*i*_) parameters, which brings us closer to the social reality, in which we do not usually have data on the strength with which the unit affects other units. Simulations have been carried out when actors have a choice of two or three opinions on a given topic. First, the spatial distribution of opinions after thousand steps of simulation was analysed. The simulations showed how clusters of opinion are formed depending on (i) the flow of information in the agents’ network, and (ii) the randomness in forming the opinion. For both *K* = 2 and *K* = 3 we can see consensus, polarisation of opinions and the formation of many clusters of available opinions.

As it was shown in previous sections, the clustering of opinions is influenced by both the level of randomness in actors’ decisions (noise) and the impact coming from neighbours. Generally, the size of the largest cluster of opinions decreases with the increase of *α* (as can be seen by inspection of columns in Figs 1 and 2). Furthermore, the number of clusters for both *K* = 2 and *K* = 3 increases with *α*, i.e. the smaller effective range of actors’ interaction the more difficult forming clusters of opinions. Intuitively, an increase in the number of clusters with an increase in noise level *T* is expected. In fact, the number of clusters is growing, but for *α* = 3 and *T* = 3 we have one cluster and single agents with opposite opinions, as can be seen in Figs 1 and 2. In this case, introducing of noise (*T*) leads to consensus with single representatives of the opposite opinion(s). In addition, for *α* ≥ 3, a slight increase of noise level (*T* = 2) induces more order than for *T* = 1 (with the exception for *α* = 6).

In addition, for *α* ≥ 3 a change in the shape of the histograms is observed. This effect is enhanced by *α* and *T*, i.e. the larger *α* the smaller *T* is needed for the shapes of the distribution to suddenly change from power-law to other, which was discussed in the ‘Clustering of opinions’ section. This change in the shape of the distribution is especially visible for *α* = 6—when the interaction effectively takes place only among near neighbours, which is shown in Fig 5. Such a noise induced transition is typical of many complex systems at the edge of chaos (see for example Ref. [76] and references therein). The consequences of this histogram shape change are also well visible in Fig 7a, 7b and 7c, where a sudden decrease in the average largest cluster size $\langle S_{\max}\rangle$, together with a sudden increase in the average number of both clusters 〈*n*_*c*_〉 and small clusters 〈*n*_*s*_〉, can be observed for *α* = 6 at *T* = 3. Moreover, the same effect is also observed for *α* = 4 and *α* = 5 (and even for *α* = 3 but for *K* = 3). In principle—for high enough *α* (when range of actors’ interactions is effectively restricted to near neighbours) and high enough level of noise *T* (when system is in the disordered phase)—the critical noise level above which histograms of opinion clusters’ sizes loose their scale-free character increases with increase of the easy of information flow. This interesting effect has been overlooked in Ref. [16] due to homogeneous initial conditions with ∀*i*: *ξ*_*i*_(*t* = 0) = Ξ_1_ applied there.

In summary, the simulations showed that opinion formation and spread is influenced by both: efficiency of information flow among actors and noise level. Better information flow, i.e. better contacts among actors facilitates the spread of opinion and its formation. In the case of small values of *α* (when information flow is very good) the unanimity of opinion is reached and consensus takes place, as in most sociophysical models of opinion dynamics [38], for both, two and three opinions available in the system. For large values of *α*—when effectively only the nearest neighbours exert impact on given actor—the polarisation of opinions is weak and there are many small groups of actors with the same opinion.

The lack of consensus in models is mainly caused by the introduction of noise [77] or anti-conformism [78]. In the presented model there is no global agreement also for *T* = 0 (when there is no noise). For *T* = 0 and *α* ≥ 3 clusters of both opinions (or three for *K* = 3) appear. In addition, in the presented model, noise for certain values of *α* promotes unanimity. This situation occurs for *α* = 3 (both for *K* = 2 and *K* = 3), when the system from the frozen state, with increasing noise *T*, achieves the consensus state for *T* = 3, before disordering for *T* = 5.

As it was mentioned earlier, many studies indicate irrationality and unpredictability in the process of forming opinions [9–13]. As our simulations have shown, this randomness in adopting opinions (noise) plays a crucial role. A low level of noise (low *T* values) results in less clusters of opinion than in the absence of noise (*T* = 0). However, the most interesting is the fact, that the high noise levels (*T* = 2, 3, 4) results in a more ordered system than for small values of noise *T* (this is the case with *α* = 3). Thus, noise favours consensus and polarisation of opinion in groups, but only when the influence of distant neighbours is significant. If the exchange of opinions takes place effectively only with near neighbours, this effect is not observed. Instead, the ‘thermal’ evolution leads system through a miscellaneous faces of disorder including:

- edges of self-organised criticality [79], where still survive some kind of long-range correlation and sizes of the clusters S vary from size of a single site to the size of whole system, what results in scale-free probability distribution functions P(S) (see Fig 5);
- a true disordered phase (where long-range correlations are definitely destroyed).

In future research, we intend to take into account the impact of strong leaders on the opinion dynamics. Also the influence of external sources of information (for instance the impact of mass media) is worth of investigation.

## Supporting information

S1 File Supporting information. (PDF)
