# Supplementary material for: Noise induced unanimity and disorder in opinion formation
Source: PLoS One. 2020 Jul 9;15(7):e0235313. doi: 10.1371/journal.pone.0235313 (PMC7347143; doi:10.1371/journal.pone.0235313)
Supplement: S1 File — (PDF) [file pone.0235313.s001.pdf]

Supporting information

Example of small system evolution ($L=3$, $K=3$) To better explain the model rules we calculate social impact on single actor for case of small lattice ($L=3$). We assume $K=3$ opinions available in the system marked as 'red' (Ξ_1), 'blue' (Ξ_2) and 'green' (Ξ_3). We will calculate the impact exerting by nine actors on the actors labelled as '5' and '9' in Fig. 8. We assume the supportiveness $s_i = i/10$ and persuasiveness $p_i = 1 - i/10$.

According to Eq. (2) to evaluate the opinion $\xi_5(t+1)$ in the next time step we have to calculated $K=3$ impacts exerted on actor $i=5$ for three opinions available in the system.

As $\xi_5(t) = \Xi_2$ ('blue') we use Eq. (2a) to calculate impact

$$I_{5,\text{blue}}(t) = 4\mathcal{J}_s\left(\frac{q(s_5)}{g(d_{5,5})} + \frac{q(s_6)}{g(d_{5,6})} + \frac{q(s_9)}{g(d_{5,9})}\right), \tag{5}$$

from all actors with 'blue' opinions (i.e. for $i = 6, 9$), including actor $i=5$ himself/herself. The impacts from actors with 'red' and 'green' opinions are calculated basing on Eq. (2b):

$$I_{5,\text{red}}(t) = 4\mathcal{J}_p\left(\frac{q(p_1)}{g(d_{5,1})} + \frac{q(p_3)}{g(d_{5,3})} + \frac{q(p_4)}{g(d_{5,4})} + \frac{q(p_7)}{g(d_{5,7})}\right), \tag{6}$$

$$I_{5,\text{green}}(t) = 4\mathcal{J}_p\left(\frac{q(p_2)}{g(d_{5,2})} + \frac{q(p_8)}{g(d_{5,8})}\right), \tag{7}$$

Fig 8. (Colour online) Example of small lattice with nine actors and three opinions. The numbers are actors labels i. The colours correspond to various actors opinions ('red'—Ξ_1, 'blue'—Ξ_2 and 'green'—Ξ_3).

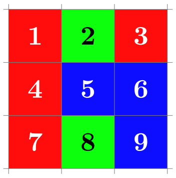

We assume identity function for scaling functions $\mathcal{J}_S(x) \equiv x$, $\mathcal{J}_P(x) \equiv x$, $q(x) \equiv x$ and the distance scaling function $g(x) = 1 + x^\alpha$, with $\alpha = 2$. These assumptions yield

$$\begin{aligned} I_{5,\text{blue}}(t) &= 4\left(\frac{s_5}{1+d_{5,5}^2} + \frac{s_6}{1+d_{5,6}^2} + \frac{s_9}{1+d_{5,9}^2}\right) \\ &= 4\left(\frac{0.5}{1+0^2} + \frac{0.6}{1+1^2} + \frac{0.9}{1+\sqrt{2}^2}\right) = 4.4, \end{aligned} \tag{8}$$

$$\begin{aligned} I_{5,\text{red}}(t) &= 4\left(\frac{p_1}{1+d_{5,1}^2} + \frac{p_3}{1+d_{5,3}^2} + \frac{p_4}{1+d_{5,4}^2} + \frac{p_7}{1+d_{5,7}^2}\right) \\ &= 4\left(\frac{0.9}{1+\sqrt{2}^2} + \frac{0.7}{1+\sqrt{2}^2} + \frac{0.6}{1+1^2} + \frac{0.3}{1+\sqrt{2}^2}\right) = 7.(3), \end{aligned} \tag{9}$$

$$I_{5,\text{green}}(t) = 4\left(\frac{p_2}{1+d_{5,2}^2} + \frac{p_8}{1+d_{5,8}^2}\right) = 4\left(\frac{0.8}{1+1^2} + \frac{0.2}{1+1^2}\right) = 2. \tag{10}$$

For $T = 0$ the largest impact on actor $i = 5$ is excreted by 'red' actors and thus—according to Eq. (1)—actor $i = 5$ in the next time step **will change** his/her opinion from 'blue' ($\xi_5(t) = \Xi_2$) to 'red' ($\xi_5(t+1) = \Xi_1$).

For $T > 0$ we calculate probabilities $P_{5,\text{blue}}$, $P_{5,\text{red}}$ and $P_{5,\text{green}}$ of choosing opinion by actor $i = 5$ (see Eqs. (4a)–(4b)). For example, for $T = 1$ these probabilities are

$$\begin{aligned} P_{5,\text{blue}} &= \frac{\exp(I_{5,\text{blue}}/1)}{P_1}, \\ P_{5,\text{red}} &= \frac{\exp(I_{5,\text{red}}/1)}{P_1}, \\ P_{5,\text{green}} &= \frac{\exp(I_{5,\text{green}}/1)}{P_1}, \end{aligned} \tag{11}$$

while for $T = 10$ we have

$$\begin{aligned} P_{5,\text{blue}} &= \frac{\exp(I_{5,\text{blue}}/10)}{P_{10}}, \\ P_{5,\text{red}} &= \frac{\exp(I_{5,\text{red}}/10)}{P_{10}}, \\ P_{5,\text{green}} &= \frac{\exp(I_{5,\text{green}}/10)}{P_{10}}, \end{aligned} \tag{12}$$

where normalisation constants are

$$P_1 = \exp(I_{5,\text{blue}}/1) + \exp(I_{5,\text{red}}/1) + \exp(I_{5,\text{green}}/1)$$

and

$$P_{10} = \exp(I_{5,\text{blue}}/10) + \exp(I_{5,\text{red}}/10) + \exp(I_{5,\text{green}}/10).$$

The calculated probabilities for $T = 1$ are

$$\begin{aligned} P_{5,\text{blue}} &= \frac{\exp(4.4/1)}{e^{4.4} + e^{7.(3)} + e^2} \approx 0.050, \\ P_{5,\text{red}} &= \frac{\exp(7.(3)/1)}{e^{4.4} + e^{7.(3)} + e^2} \approx 0.945, \\ P_{5,\text{green}} &= \frac{\exp(2/1)}{e^{4.4} + e^{7.(3)} + e^2} \approx 0.005, \end{aligned} \tag{13}$$

while for $T = 10$ we have

$$\begin{aligned} P_{5,\text{blue}} &= \frac{\exp(4.4/10)}{e^{0.44} + e^{0.7(3)} + e^{0.2}} \approx 0.320, \\ P_{5,\text{red}} &= \frac{\exp(7.(3)/10)}{e^{0.44} + e^{0.7(3)} + e^{0.2}} \approx 0.429, \\ P_{5,\text{green}} &= \frac{\exp(2/10)}{e^{0.44} + e^{0.7(3)} + e^{0.2}} \approx 0.251. \end{aligned} \tag{14}$$

For non-deterministic version of algorithm (i.e. for $T > 0$) still the most probably state $\xi_5(t+1)$ is Ξ_1 ('red'). But probability of such evolution for actor $i = 5$ decreases from 100% for $T = 0$ to 94.5% for $T = 1$ and to 42.9% for $T = 10$ to become 33.3%$= 1/K$ for $T \to \infty$.

Let us repeat these calculation for actor $i = 9$:

$$I_{9,\text{blue}}(t) = 4\mathcal{J}_s \left(\frac{q(s_5)}{g(d_{9,5})} + \frac{q(s_6)}{g(d_{9,6})} + \frac{q(s_9)}{g(d_{9,9})} \right), \tag{15}$$

$$I_{9,\text{red}}(t) = 4\mathcal{J}_p\left(\frac{q(p_1)}{g(d_{9,1})} + \frac{q(p_3)}{g(d_{9,3})} + \frac{q(p_4)}{g(d_{9,4})} + \frac{q(p_7)}{g(d_{9,7})}\right), \tag{16}$$

$$I_{9,\text{green}}(t) = 4\mathcal{J}_p\left(\frac{q(p_2)}{g(d_{9,2})} + \frac{q(p_8)}{g(d_{9,8})}\right), \tag{17}$$

$$\begin{aligned} I_{9,\text{blue}}(t) &= 4\left(\frac{s_5}{1+d_{9,5}^2} + \frac{s_6}{1+d_{9,6}^2} + \frac{s_9}{1+d_{9,9}^2}\right) \\ &= 4\left(\frac{0.5}{1+\sqrt{2}^2} + \frac{0.6}{1+1^2} + \frac{0.9}{1+0^2}\right) = 5.4(6), \end{aligned} \tag{18}$$

$$\begin{aligned} I_{9,\text{red}}(t) &= 4\left(\frac{p_1}{1+d_{9,1}^2} + \frac{p_3}{1+d_{9,3}^2} + \frac{p_4}{1+d_{9,4}^2} + \frac{p_7}{1+d_{9,7}^2}\right) \\ &= 4\left(\frac{0.9}{1+(2\sqrt{2})^2} + \frac{0.7}{1+2^2} + \frac{0.6}{1+\sqrt{5}^2} + \frac{0.3}{1+2^2}\right) = 1.6, \end{aligned} \tag{19}$$

$$I_{9,\text{green}}(t) = 4\left(\frac{p_2}{1+d_{9,2}^2} + \frac{p_8}{1+d_{9,8}^2}\right) = 4\left(\frac{0.8}{1+\sqrt{5}^2} + \frac{0.2}{1+1^2}\right) = 0.9(3). \tag{20}$$

For $T = 0$ the largest impact on actor $i = 9$ is excreted by ‘blue’ actors and thus—according to Eq. (1)—actor $i = 9$ in the next time step **will sustain** his/her ‘blue’ opinion ($\xi_9(t+1) = \xi_9(t) = \Xi_2$). Two factors influence the difference in actors $i = 5$ and $i = 9$ opinion in time $(t+1)$. Namely, the difference in supportiveness of these two actors and their distance to ‘red’ actors: actor $i = 5$ has moderate supportiveness ($s_5 = 0.5$) and his/her distance to ‘red’ actors is no longer than $\sqrt{2}$. In contrast, actor $i = 9$ has very high supportiveness ($s_9 = 0.9$) and distance to ‘red’ actors no shorter than 2. Please note however, that ultimate fate of the system is the state with the unanimity of opinions. As we have shown above, in the next time step at least the actor in the middle of the system ($i = 5$) will convert his/her opinion to the ‘red’ one. The same presumably will occur for actor $i = 2$ who has low supportiveness ($s_2 = 0.2$) and who has only a single supporter. Thus in time $(t+3)$ all actors will convert to the supporters of the ‘red’ opinion.

For $T > 0$ we calculate probabilities $P_{9,\text{blue}}$, $P_{9,\text{red}}$ and $P_{9,\text{green}}$ of choosing opinion by actor $i = 9$ (see Eqs. (4a)–(4b)). For example, for $T = 1$ these probabilities are

$$\begin{aligned} P_{9,\text{blue}} &= \frac{\exp(I_{9,\text{blue}}/1)}{P_1}, \\ P_{9,\text{red}} &= \frac{\exp(I_{9,\text{red}}/1)}{P_1}, \\ P_{9,\text{green}} &= \frac{\exp(I_{9,\text{green}}/1)}{P_1}, \end{aligned} \tag{21}$$

while for $T = 10$ we have

$$\begin{aligned} P_{9,\text{blue}} &= \frac{\exp(I_{9,\text{blue}}/10)}{P_{10}}, \\ P_{9,\text{red}} &= \frac{\exp(I_{9,\text{red}}/10)}{P_{10}}, \\ P_{9,\text{green}} &= \frac{\exp(I_{9,\text{green}}/10)}{P_{10}}, \end{aligned} \tag{22}$$

Table 1. Histogram of cluster sizes $\mathcal{S}$ for lattices presented in Fig. 9.

labels i:	6, 9, 11, 15	3	8, 12	10	2	4, 7	1	5	13	14
$\mathcal{S}$:	1	2	3	5	8	14	25	26	42	54
$n(\mathcal{S})$:	4	1	2	1	1	2	1	1	1	1

where normalisation constants are

$$P_1 = \exp(I_{9,\text{blue}}/1) + \exp(I_{9,\text{red}}/1) + \exp(I_{9,\text{green}}/1)$$

and

$$P_{10} = \exp(I_{9,\text{blue}}/10) + \exp(I_{9,\text{red}}/10) + \exp(I_{9,\text{green}}/10).$$

The calculated probabilities for $T = 1$ are

$$\begin{aligned} P_{9,\text{blue}} &= \frac{\exp(5.4(6)/1)}{e^{5.4(6)} + e^{1.6} + e^{0.9(3)}} \approx 0.969, \\ P_{9,\text{red}} &= \frac{\exp(1.6/1)}{e^{5.4(6)} + e^{1.6} + e^{0.9(3)}} \approx 0.020, \\ P_{9,\text{green}} &= \frac{\exp(0.9(3)/1)}{e^{5.4(6)} + e^{1.6} + e^{0.9(3)}} \approx 0.011, \end{aligned} \tag{23}$$

while for $T = 10$ we have

$$\begin{aligned} P_{9,\text{blue}} &= \frac{\exp(5.4(6)/10)}{e^{0.54(6)} + e^{0.16} + e^{0.09(3)}} \approx 0.432, \\ P_{9,\text{red}} &= \frac{\exp(1.6/10)}{e^{0.54(6)} + e^{0.16} + e^{0.09(3)}} \approx 0.293, \\ P_{9,\text{green}} &= \frac{\exp(0.9(3)/10)}{e^{0.54(6)} + e^{0.16} + e^{0.09(3)}} \approx 0.275. \end{aligned} \tag{24}$$

Similarly to the actor $i = 5$, the increase of the social temperature reduces chance of keeping initial opinion for actor $i = 9$. For $T = 10$ these probabilities do not differ from $1/K$ for more than 0.1.

Small example of clustering ($L = 10$, $K = 3$) Two sites are in the same cluster if they are adjacent (in von Neumann neighbourhood) to each other and simultaneously actors at these sites share the same opinion. The Hoshen–Kopelman algorithm allows for sites labelling in such way that sites in the same cluster have the same labels and sites in different cluster have different labels. Examples of sites labelling for $L = 10$ and $K = 3$ are presented in Figs. 9a and 9b, where $n_c = 11$ and $n_c = 4$ clusters have been identified, respectively. The average number of cluster for these two lattice realisation is $\langle n_c \rangle = (11 + 4)/2 = 7.5$. The number of sites in each cluster defines its size $\mathcal{S}$. For these two lattice realisations the largest clusters are labelled as 5 (Fig. 9a) and as 14 (Fig. 9b) and their sizes are $\mathcal{S}_{\max} = 26$ and $\mathcal{S}_{\max} = 54$, respectively. Thus average largest cluster size is $\langle \mathcal{S}_{\max} \rangle = (26 + 54)/2 = 40$. In given example histogram $H(\mathcal{S})$ of clusters sizes is presented in Table 1. Basing on Table 1 we evaluate number of small clusters (with $\mathcal{S} \le 5$) as 4+1+2+1=8. As this sum comes from merging results of two lattice realisation the average number of small clusters is $\langle n_s \rangle = 8/2 = 4$.

Source codes In Listings 1 and 2 the Fortran 95 codes allowing for reproductions of data for Figs. 3, 4, 6, 7 (for both, noiseless and non-deterministic version of simulations) are presented.

Fig 9. Example of sites labelling for $K = 3$ and $L = 10$ and two lattice realisations.

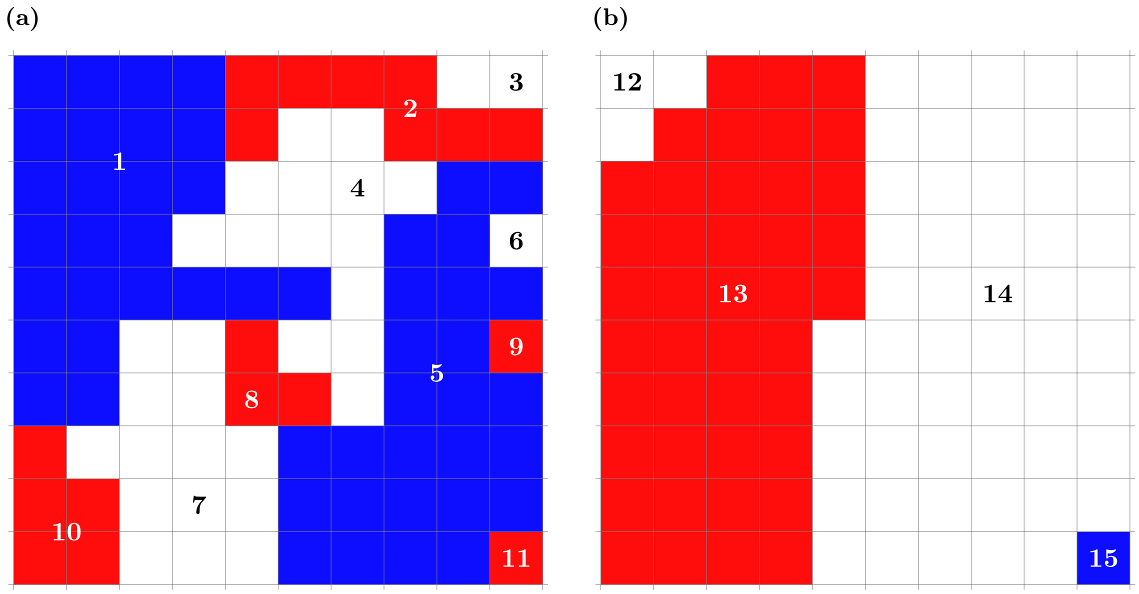

The module `settings` provides model parameters including lattice size L (`Xmax` and `Ymax`), number of opinions K (`Kmax`), number of time steps $t_{\max}$ (`Tmax`) and number of lattice realisations (`Run`).

In module `utils` the scaling functions $g(x)$ and $q(x)$ as well as the Euclidean distance $d(x, y)$ are defined. Also the `reclassify` function for Hoshen–Kopelman algorithm is defined there.

The main program starts in line 52. The actors supportiveness (s_i) and persuasiveness (p_i) are initialised randomly in lines 85–90, while initial actors opinions (ξ_i) are given in lines 93–98. Loop 88 provides time evolution of the system. Loop 77 realises Hoshen–Kopelman algorithm of sites (actors) labelling for $t = 0$. In loop 99 the system characteristics after the system time evolution is completed are calculated. Loop 777 realises averaging procedure over independent simulations for various initial conditions.

Deterministic version, $T = 0$ An input data (α parameter) is read in line 71. In lines 272–275 histograms $H(\mathcal{S})$ of clusters sizes $\mathcal{S}$ are printed. In line 278 values of $\langle n_c \rangle$, $\langle S \rangle$ (not presented in this paper) and $\langle \mathcal{S}_{\max} \rangle$ are printed.

Listing 1. Fortan95 code implementing Eq. (1) i.e. for $T = 0$

```
1  !!! Latane-Nowak-Szamrej model + Hoshen-Kopelman algorithm
2  !!! K. Malarz
3  !!! created: Tue, 21 May 2019, 13:06:13 CEST
4  !!! revised: Sun, 05 Apr 2020, 14:43:19 CEST
5
6  !!! ==================================================================
7  module settings
8  !!! ==================================================================
9  implicit none
10
11 integer, parameter :: Xmax=41,Ymax=41,Tmax=1000,Kmax=2,L2=(Xmax+1)*(Ymax+1),Run=100
12 real*8, parameter :: T=0.d0
13 real*8 :: alpha
14 end module settings
15
```

```fortran
!!! ==================================================================
module utils
!!! ==================================================================
use settings
implicit none
contains

real*8 function g(x)
        real*8 :: x
        g=1.0d0+x**alpha
end function

real*8 function q(x)
        real*8 :: x
        q=x
end function

real*8 function d(x1,y1,x2,y2)
        integer :: x1,y1,x2,y2
        d=dsqrt((1.d0*x1-1.d0*x2)**2 + (1.d0*y1-1.d0*y2)**2)
end function

integer function reclassify(ix)
integer :: ix
integer, dimension (0:Xmax,0:Ymax) :: label
integer, dimension (L2) :: iclass
common/block/ label, iclass

reclassify=iclass(ix)
90 if(iclass(reclassify).eq.reclassify) return
reclassify=iclass(reclassify)
goto 90
end function

end module utils

!!! ##################################################################
program Latane_Hoshen_Kopelman
!!! ##################################################################
use settings
use utils
implicit none
integer :: x,y,it, xx,yy,k,kk,strongest_k,irun,maxlabel,Smax,largestS
real :: r
real*8 :: sump,strongest_I,avenc,aveS,aveSmax

integer, dimension (0:Xmax,0:Ymax) :: label
integer, dimension (L2) :: iclass
integer, dimension (0:Xmax,0:Ymax) :: xi
integer, dimension (0:Xmax*Ymax) :: isize, histograminrun,histogram
integer, dimension (0:Xmax*Ymax,Kmax) :: histogramK
real*8, dimension (Xmax,Ymax) :: p, s
real*8, dimension (Xmax,Ymax,Kmax) :: I, prob
common/block/ label, iclass

  read *,alpha
  print '(A3,7A11)','###','Xmax','Ymax','K','alpha','T','tmax','run'
  print '(A3,3I11,2F11.3,2I11)','###',Xmax,Ymax,Kmax,alpha,T,Tmax,Run

  histogram=0
  aveSmax=0.d0
  aveS=0.d0
  avenc=0.d0

  do 777 irun=1,Run
  histograminrun=0
```

```
!   print *,'#########'
!   print *,'# irun=',irun

  do x=1,Xmax !! initial state
  do y=1,Ymax
    s(x,y)=rand()
    p(x,y)=rand()
  enddo
  enddo

  it=0
  xi=0
  do x=1,Xmax
  do y=1,Ymax
    xi(x,y)=1+Kmax*rand()
  enddo
  enddo

  histogramK=0

  do 77 k=1,Kmax
    isize=0

    label=L2
    do kk=1,L2
      iclass(kk)=kk
    enddo
    maxlabel=0

    do x=1,Xmax
    do y=1,Ymax
      if(xi(x,y).eq.k) then !! labeling clusters
        if(xi(x-1,y).eq.k .or. xi(x,y-1).eq.k) then
          !! reclassifing neighbouring sites
          if(xi(x-1,y).eq.k) label(x-1,y)=reclassify(label(x-1,y))
          if(xi(x,y-1).eq.k) label(x,y-1)=reclassify(label(x,y-1))
          label(x,y)=min(label(x-1,y),label(x,y-1))
          iclass(label(x-1,y))=label(x,y)
          iclass(label(x,y-1))=label(x,y)
        else
          maxlabel=maxlabel+1
          label(x,y)=maxlabel
        endif
      endif
    enddo
    enddo
    ! reclassifing all occupied sites
    do x=1,Xmax
    do y=1,Ymax
      if((xi(x,y).eq.k) .and. (label(x,y).gt.iclass(label(x,y)))) label(x,y)=reclassify(label(x,y))
    enddo
    enddo

    do x=1,Xmax
    do y=1,Ymax
      if(xi(x,y).eq.k) isize(label(x,y))=isize(label(x,y))+1
    enddo
    enddo

    do kk=1,Xmax*Ymax
      histogramK(isize(kk),k)=histogramK(isize(kk),k)+1
    enddo

77 enddo

   do 88 it=1,Tmax !!! time evolution
```

```
      I=0.0d0
      do x=1,Xmax
      do y=1,Ymax
        do xx=1,Xmax
        do yy=1,Ymax
          if(xi(x,y).eq.xi(xx,yy)) then
            I(x,y,xi(xx,yy))=I(x,y,xi(xx,yy))+q(s(xx,yy))/g(d(x,y,xx,yy))
          else
            I(x,y,xi(xx,yy))=I(x,y,xi(xx,yy))+q(p(xx,yy))/g(d(x,y,xx,yy))
          endif
        enddo
        enddo
      enddo
      enddo

      do x=1,Xmax
      do y=1,Ymax
      do k=1,Kmax
        I(x,y,k)=4.0d0*I(x,y,k)
      enddo
      enddo
      enddo

      do x=1,Xmax
      do y=1,Ymax
        strongest_I=I(x,y,1)
        strongest_k=1
        do k=2,Kmax
          if(I(x,y,k).gt.strongest_I) then
            strongest_I=I(x,y,k)
            strongest_k=k
          endif
        enddo
        xi(x,y)=strongest_k
      enddo
      enddo
88 enddo !!! time evolution

!    print *,'# it=',it,'xi:'
!    do x=1,Xmax
!       print '(41I5)',(xi(x,y),y=1,Ymax)
!    enddo
   histogramK=0
   Smax=0

   do 99 k=1,Kmax
     isize=0

!     print *,"# k=",k
     label=L2
     do kk=1,L2
       iclass(kk)=kk
     enddo
     maxlabel=0

     do x=1,Xmax
     do y=1,Ymax
       if(xi(x,y).eq.k) then ! labeling clusters
         if(xi(x-1,y).eq.k .or. xi(x,y-1).eq.k) then
           ! reclassifing neighbouring sites
           if(xi(x-1,y).eq.k) label(x-1,y)=reclassify(label(x-1,y))
           if(xi(x,y-1).eq.k) label(x,y-1)=reclassify(label(x,y-1))
           label(x,y)=min(label(x-1,y),label(x,y-1))
           iclass(label(x-1,y))=label(x,y)
           iclass(label(x,y-1))=label(x,y)
         else
```

```fortran
          maxlabel=maxlabel+1
          label(x,y)=maxlabel
        endif
      endif
    enddo
    enddo
    ! reclassifing all occupied sites
    do x=1,Xmax
    do y=1,Ymax
      if((xi(x,y).eq.k) .and. (label(x,y).gt.iclass(label(x,y)))) label(x,y)=reclassify(label(x,y))
    enddo
    enddo

!     do x=1,Xmax
!       print '(41I5)',(label(x,y),y=1,Ymax)
!     enddo

    do x=1,Xmax
    do y=1,Ymax
      if(xi(x,y).eq.k) isize(label(x,y))=isize(label(x,y))+1
    enddo
    enddo

    do kk=1,Xmax*Ymax
      histogramK(isize(kk),k)=histogramK(isize(kk),k)+1
    enddo

!     print *,"# histogram, irun=",irun," k=",k
    do kk=1,Xmax*Ymax
!       if(histogramK(kk,k).gt.0) print *,kk,histogramK(kk,k)
      histograminrun(kk)=histograminrun(kk)+histogramK(kk,k)
    enddo

    do kk=Xmax*Ymax,1,-1
      if(histogramK(kk,k).gt.0) then
        largestS=kk
        goto 33
      endif
    enddo
33  Smax=max(Smax,largestS)
!   print *,"# largest S=",largestS
!   print *,"# Smax=",Smax

99  enddo

!   do k=1,Xmax*Ymax
!     if(histograminrun(k).gt.0) print *,k,histograminrun(k)
!   enddo
!   print *,"# nc=",sum(histograminrun)
    avenc=avenc+1.d0*sum(histograminrun)
    aveSmax=aveSmax+1.d0*Smax
    do k=1,Xmax*Ymax
      aveS=aveS+(1.d0*k*histograminrun(k))/(1.d0*sum(histograminrun))
      histogram(k)=histogram(k)+histograminrun(k)
    enddo

777 enddo

  print *,"# total histogram:"
  do k=1,Xmax*Ymax
    if(histogram(k).gt.0) print *,k,histogram(k)
  enddo

  print '(A2,A3,5A9)',"#","K","T","alpha","<nc>","<S>","<Smax>"
  print '(A2,I3,5F9.3)',"#",Kmax,T,alpha,avenc/(1.d0*Run),aveS/(1.d0*Run),aveSmax/(1.d0*Run)

```

```
280 end program Latane_Hoshen_Kopelman
```

Probabilistic version, $T > 0$ An input data (α and T parameters) are read in line 71. In lines 284–287 histograms $H(\mathcal{S})$ of clusters size $\mathcal{S}$ are printed. In line 290 values of $\langle n_c \rangle$, $\langle S \rangle$ (not presented in this paper), $\langle \mathcal{S}_{\max} \rangle$ are printed.

Listing 2. Fortan95 code implementing Eq. (4) i.e. for $T > 0$

```
1  !!! Latane-Nowak-Szamrej model + Hoshen-Kopelman algorithm
2  !!! K. Malarz
3  !!! created: Tue, 21 May 2019, 13:06:13 CEST
4  !!! revised: Sun, 05 Apr 2020, 14:43:19 CEST
5
6  !!! ====================================================================
7  module settings
8  !!! ====================================================================
9  implicit none
10
11 integer, parameter :: Xmax=41,Ymax=41,Tmax=1000,Kmax=2,L2=(Xmax+1)*(Ymax+1),Run=100
12 real*8 :: T
13 real*8 :: alpha
14 end module settings
15
16 !!! ====================================================================
17 module utils
18 !!! ====================================================================
19 use settings
20 implicit none
21 contains
22
23 real*8 function g(x)
24         real*8 :: x
25         g=1.0d0+x**alpha
26 end function
27
28 real*8 function q(x)
29         real*8 :: x
30         q=x
31 end function
32
33 real*8 function d(x1,y1,x2,y2)
34         integer :: x1,y1,x2,y2
35         d=dsqrt((1.d0*x1-1.d0*x2)**2 + (1.d0*y1-1.d0*y2)**2)
36 end function
37
38 integer function reclassify(ix)
39 integer :: ix
40 integer, dimension (0:Xmax,0:Ymax) :: label
41 integer, dimension (L2) :: iclass
42 common/block/ label, iclass
43
44 reclassify=iclass(ix)
45 90 if(iclass(reclassify).eq.reclassify) return
46 reclassify=iclass(reclassify)
47 goto 90
48 end function
49
50 end module utils
51
52 !!! ####################################################################
53 program Latane_Hoshen_Kopelman
54 !!! ####################################################################
55 use settings
56 use utils
```

```fortran
implicit none
integer :: x,y,it, xx,yy,k,kk,strongest_k,irun,maxlabel,Smax,largestS
real :: r
real*8 :: sump,avenc,aveS,aveSmax

integer, dimension (0:Xmax,0:Ymax) :: label
integer, dimension (L2) :: iclass
integer, dimension (0:Xmax,0:Ymax) :: xi
integer, dimension (0:Xmax*Ymax) :: isize, histograminrun,histogram
integer, dimension (0:Xmax*Ymax,Kmax) :: histogramK
real*8, dimension (Xmax,Ymax) :: p, s
real*8, dimension (Xmax,Ymax,Kmax) :: I, prob
common/block/ label, iclass

  read *,T,alpha
  print '(A3,7A11)','###','Xmax','Ymax','K','alpha','T','tmax','run'
  print '(A3,3I11,2F11.3,2I11)','###',Xmax,Ymax,Kmax,alpha,T,Tmax,Run

  histogram=0
  aveSmax=0.d0
  aveS=0.d0
  avenc=0.d0

  do 777 irun=1,Run
  histograminrun=0
!  print *,'#########'
!  print *,'# irun=',irun

  do x=1,Xmax !! initial state
  do y=1,Ymax
    s(x,y)=rand()
    p(x,y)=rand()
  enddo
  enddo

  it=0
  xi=0
  do x=1,Xmax
  do y=1,Ymax
    xi(x,y)=1+Kmax*rand()
  enddo
  enddo

  histogramK=0

  do 77 k=1,Kmax
    isize=0

    label=L2
    do kk=1,L2
      iclass(kk)=kk
    enddo
    maxlabel=0

    do x=1,Xmax
    do y=1,Ymax
      if(xi(x,y).eq.k) then !! labeling clusters
        if(xi(x-1,y).eq.k .or. xi(x,y-1).eq.k) then
          !! reclassifing neighbouring sites
          if(xi(x-1,y).eq.k) label(x-1,y)=reclassify(label(x-1,y))
          if(xi(x,y-1).eq.k) label(x,y-1)=reclassify(label(x,y-1))
          label(x,y)=min(label(x-1,y),label(x,y-1))
          iclass(label(x-1,y))=label(x,y)
          iclass(label(x,y-1))=label(x,y)
        else
          maxlabel=maxlabel+1
```

```
          label(x,y)=maxlabel
        endif
      endif
    enddo
    enddo
    ! reclassifing all occupied sites
    do x=1,Xmax
    do y=1,Ymax
      if((xi(x,y).eq.k) .and. (label(x,y).gt.iclass(label(x,y)))) label(x,y)=reclassify(label(x,y))
    enddo
    enddo

    do x=1,Xmax
    do y=1,Ymax
      if(xi(x,y).eq.k) isize(label(x,y))=isize(label(x,y))+1
    enddo
    enddo

    do kk=1,Xmax*Ymax
      histogramK(isize(kk),k)=histogramK(isize(kk),k)+1
    enddo

77 enddo

   do 88 it=1,Tmax !!! time evolution
     I=0.0d0
     do x=1,Xmax
     do y=1,Ymax
       do xx=1,Xmax
       do yy=1,Ymax
          if(xi(x,y).eq.xi(xx,yy)) then
            I(x,y,xi(xx,yy))=I(x,y,xi(xx,yy))+q(s(xx,yy))/g(d(x,y,xx,yy))
          else
            I(x,y,xi(xx,yy))=I(x,y,xi(xx,yy))+q(p(xx,yy))/g(d(x,y,xx,yy))
          endif
       enddo
       enddo
     enddo
     enddo

     do x=1,Xmax
     do y=1,Ymax
     do k=1,Kmax
       I(x,y,k)=4.0d0*I(x,y,k)
     enddo
     enddo
     enddo

     do x=1,Xmax
     do y=1,Ymax
       sump=0.0d0
       do k=1,Kmax
          prob(x,y,k)=dexp(I(x,y,k)/T)
          sump=sump+prob(x,y,k)
       enddo
       do k=1,Kmax
          prob(x,y,k)=prob(x,y,k)/sump
       enddo
     enddo
     enddo

     do x=1,Xmax
     do y=1,Ymax
       r=rand()
       sump=0.0d0
       do k=1,Kmax
```

```
            sump=sump+prob(x,y,k)
            if(r.lt.sump) goto 666
         enddo
666      xi(x,y)=k
      enddo
      enddo

88 enddo !!! time evolution

!    print *,'# it=',it,'xi:'
!    do x=1,Xmax
!       print '(41I5)',(xi(x,y),y=1,Ymax)
!    enddo
   histogramK=0
   Smax=0

   do 99 k=1,Kmax
      isize=0

!      print *,"# k=",k
      label=L2
      do kk=1,L2
         iclass(kk)=kk
      enddo
      maxlabel=0

      do x=1,Xmax
      do y=1,Ymax
         if(xi(x,y).eq.k) then ! labeling clusters
            if(xi(x-1,y).eq.k .or. xi(x,y-1).eq.k) then
               ! reclassifing neighbouring sites
               if(xi(x-1,y).eq.k) label(x-1,y)=reclassify(label(x-1,y))
               if(xi(x,y-1).eq.k) label(x,y-1)=reclassify(label(x,y-1))
               label(x,y)=min(label(x-1,y),label(x,y-1))
               iclass(label(x-1,y))=label(x,y)
               iclass(label(x,y-1))=label(x,y)
            else
               maxlabel=maxlabel+1
               label(x,y)=maxlabel
            endif
         endif
      enddo
      enddo
      ! reclassifing all occupied sites
      do x=1,Xmax
      do y=1,Ymax
         if((xi(x,y).eq.k) .and. (label(x,y).gt.iclass(label(x,y)))) label(x,y)=reclassify(label(x,y))
      enddo
      enddo

!      do x=1,Xmax
!         print '(41I5)',(label(x,y),y=1,Ymax)
!      enddo

      do x=1,Xmax
      do y=1,Ymax
         if(xi(x,y).eq.k) isize(label(x,y))=isize(label(x,y))+1
      enddo
      enddo

      do kk=1,Xmax*Ymax
         histogramK(isize(kk),k)=histogramK(isize(kk),k)+1
      enddo

!      print *,"# histogram, irun=",irun," k=",k
      do kk=1,Xmax*Ymax
```

```
!        if(histogramK(kk,k).gt.0) print *,kk,histogramK(kk,k)
           histograminrun(kk)=histograminrun(kk)+histogramK(kk,k)
       enddo

       do kk=Xmax*Ymax,1,-1
          if(histogramK(kk,k).gt.0) then
            largestS=kk
            goto 33
          endif
       enddo
33     Smax=max(Smax,largestS)
!      print *,"# largest S=",largestS
!      print *,"# Smax=",Smax

99   enddo

!      do k=1,Xmax*Ymax
!         if(histograminrun(k).gt.0) print *,k,histograminrun(k)
!      enddo
!      print *,"# nc=",sum(histograminrun)
       avenc=avenc+1.d0*sum(histograminrun)
       aveSmax=aveSmax+1.d0*Smax
       do k=1,Xmax*Ymax
          aveS=aveS+(1.d0*k*histograminrun(k))/(1.d0*sum(histograminrun))
          histogram(k)=histogram(k)+histograminrun(k)
       enddo

777  enddo

    print *,"# total histogram:"
    do k=1,Xmax*Ymax
       if(histogram(k).gt.0) print *,k,histogram(k)
    enddo

    print '(A2,A3,5A9)',"#","K","T","alpha","<nc>","<S>","<Smax>"
    print '(A2,I3,5F9.3)',"#",Kmax,T,alpha,avenc/(1.d0*Run),aveS/(1.d0*Run),aveSmax/(1.d0*Run)

end program Latane_Hoshen_Kopelman
```
